# Enhanced SIRT3 expression restores mitochondrial quality control mechanism to reverse osteogenic impairment in type 2 diabetes mellitus

**DOI:** 10.1038/s41413-024-00399-5

**Published:** 2025-03-03

**Authors:** Yansi Xian, Bin Liu, Tao Shen, Lin Yang, Rui Peng, Hongdou Shen, Xueying An, Yutian Wang, Yu Ben, Qing Jiang, Baosheng Guo

**Affiliations:** 1 Division of Sports Medicine and Adult Reconstructive Surgery, Department of Orthopedic Surgery, Nanjing Drum Tower Hospital, Affiliated Hospital of Medical School, Nanjing University, 321 Zhongshan Road, Nanjing, 210008 Jiangsu PR China; 2 State Key Laboratory of Pharmaceutical Biotechnology, Nanjing University, 22 Hankou Road, Nanjing, 210093 Jiangsu PR China; 3 Branch of National Clinical Research Center for Orthopedics, Sports Medicine and Rehabilitation, 321 Zhongshan Road, Nanjing, 210008 Jiangsu PR China; 4 Medical School of Nanjing University, 22 Hankou Road, Nanjing, 210093 Jiangsu PR China; 5 Division of Sports Medicine and Adult Reconstructive Surgery, Department of Orthopedic Surgery, Nanjing Drum Tower Hospital Clinical College of Nanjing Medical University, 321 Zhongshan Road, Nanjing, 210008 Jiangsu PR China

**Keywords:** Bone, Osteoporosis, Pathogenesis

## Abstract

Osteoporosis represents a prevalent and debilitating comorbidity in patients diagnosed with type 2 diabetes mellitus (T2DM), which is characterized by suppressed osteoblast function and disrupted bone microarchitecture. In this study, we utilized male C57BL/6 J mice to investigate the role of SIRT3 in T2DM. Decreased SIRT3 expression and impaired mitochondrial quality control mechanism are observed in both in vitro and in vivo models of T2DM. Mechanistically, SIRT3 suppression results in hyperacetylation of FOXO3, hindering the activation of the PINK1/PRKN mediated mitophagy pathway and resulting in accumulation of dysfunctional mitochondria. Genetical overexpression or pharmacological activation of SIRT3 restores deacetylation status of FOXO3, thus facilitating mitophagy and ameliorating osteogenic impairment in T2DM. Collectively, our findings highlight the fundamental regulatory function of SIRT3 in mitochondrial quality control, crucial for maintaining bone homeostasis in T2DM. These insights not only enhance our understanding of the molecular mechanisms underlying diabetic osteoporosis but also identify SIRT3 as a promising therapeutic target for diabetic osteoporosis.

## Introduction

Type 2 diabetes mellitus (T2DM), characterized by insulin resistance and reduced β-cell function, is a common metabolic disease with increasing prevalence throughout the world that causes an enormous medical and economic burden.^1,2^ Recent findings suggest that individuals with T2DM demonstrate disrupted bone microarchitecture, partly due to impaired osteoblast function.^3–5^ Despite an increased risk of fragility fractures in T2DM patients, the exact molecular mechanisms attributing to osteoblast dysfunction remain to be explored, hindering the investigation of therapeutic strategies for patients with diabetic osteoporosis.

Mitochondria plays a crucial role in cellular homeostasis, and mitochondrial damage is associated with a broad spectrum of pathologies.^6,7^ Emerging evidence has shown that mitochondrial dysfunction takes the center stage in the development and progression of diabetes and its complications such as diabetic osteoporosis.^8^ Sustained mitochondrial damage results in the accumulation of reactive oxygen species (ROS) which leads to the disorder of cell homeostasis.^6,9^ Among the mechanisms responsible for regulating mitochondrial quality, mitophagy, a selective form of autophagy, is a critical process for maintaining cellular health by removing damaged mitochondria.^10,11^ PTEN-induced kinase 1 (PINK1)- E3 ubiquitin-protein ligase parkin (PRKN) pathway has been identified as a signaling pathway that initiates mitophagy,^12^ where PINK1 accumulates on the outer mitochondrial membrane upon mitochondrial depolarization, subsequently recruiting and activating PRKN and autophagy receptors to remove impaired mitochondria.^13,14^ Recent studies have demonstrated the essential role of PINK1 in osteoblast differentiation,^15,16^ and highlighted the therapeutic potential of enhancing osteoblast mitophagy to address age-related osteoporosis.^17^ Hence, dysregulated mitophagy could potentially involves in the pathogenesis of diabetic osteoporosis; however, the mechanisms underlying the dysregulation of mitophagy in osteoblasts within the diabetic milieu are not yet fully understood.

Sirtuin family is a group of highly-conserved nicotinamide adenine dinucleotide (NAD^+^)-dependent protein deacetylases, encompassing seven members (SIRT1-SIRT7) in mammals which significantly influence cellular homeostasis.^18,19^ Among these, Sirtuin 3 (SIRT3) is a major mitochondrial deacetylase that impacting energy metabolism, antioxidant defense, and mitochondrial dynamics.^20,21^ Our prior investigation revealed that SIRT3 mediates the benefits of exercise on bones in aged mice via influencing the formation of osteocyte dendritic processes.^22^ Moreover, emerging studies have indicated that SIRT3-modulated mitophagy is involved in various diseases such as nonalcoholic fatty liver disease, viral infections, and acute kidney injury.^23–25^ Forkhead box protein O3 (FOXO3) is a transcription factor which regulates the expression of various genes and proteins.^26^ It has been reported that FOXO3 could promote mitophagy and improve cardiac function through upregulating *Prkn*.^26,27^ And SIRT3 could directly interact with FOXO3 and mediate its deacetylation, which facilitate translocation of deacetylated FOXO3 to the nucleus.^28–30^ Therefore, it is worth exploring the underlying mechanism by which SIRT3/FOXO3 axis regulates osteoblast mitophagy in T2DM.

In the present study, reduced bone mass accompanied by decreased SIRT3 expression and impaired mitophagy in osteoblasts are observed in T2DM mice. Our findings strongly confirmed that SIRT3 activation alleviates bone formation by upregulating mitophagy. Mechanistically, SIRT3 modulates the acetylation of FOXO3, subsequently facilitate its nuclear translocation and restores the mitophagy via targeting *Prkn*. These findings offer valuable insights into the pathophysiology of diabetic osteoporosis and propose a promising therapeutic target for future diabetic osteoporosis treatment.

## Results

### Suppressed osteoblast activity results in low bone mass of HFD&STZ mice

**Fig. 1:**
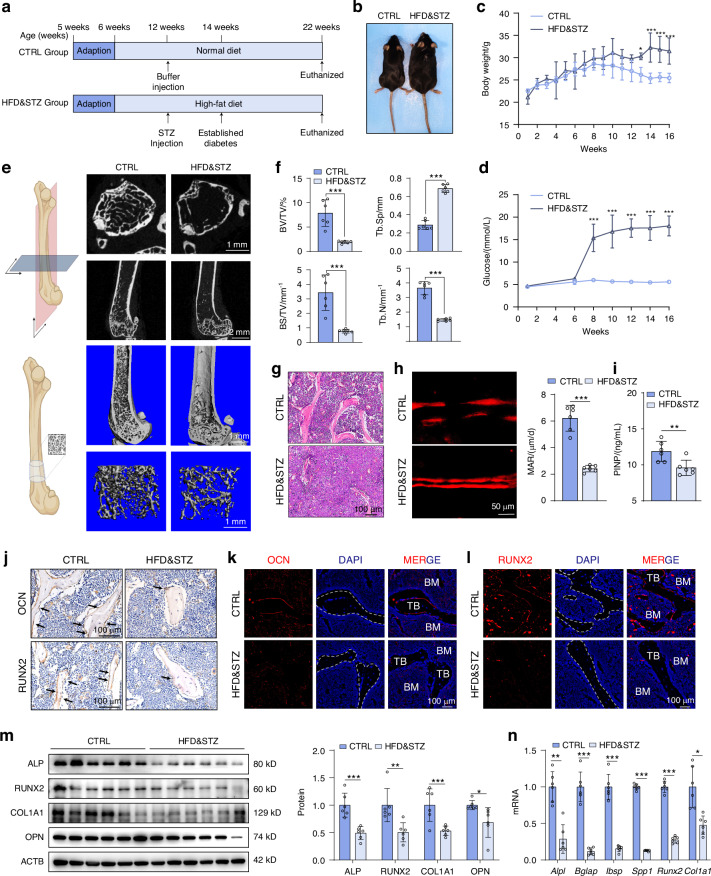
T2DM mice exhibit obvious bone loss with suppressed osteoblast activity. **a** Schematic illustration of the establishment T2DM model of male C57BL/6 J mice with HFD feeding, following 35 mg/kg STZ injection (HFD&STZ mice). **b** Representative images of CTRL and HFD&STZ mice. **c** Weekly assessment of body weight. **d** Biweekly measurement of fasting blood glucose. **e** Representative micro-CT images of femurs from CTRL and HFD&STZ mice. *n* = 6 per group. The illustrations for indicating the analysed region was created with Biorender.com. **f** Quantification of bone parameters based on micro-CT analyses, including bone volume per tissue volume (BV/TV), tracbecular spacing (Tb.Sp), bone surface per tissue volume (BS/TV) and trabecular number (Tb.N). **g** Representative images of H&E staining. Scale bar, 100 μm. **h** Representative images and quantification of xylenol orange double labeling of the femurs from CTRL and HFD&STZ mice. Scale bar, 50 μm. **i** Serum concentration of PINP. *n* = 6 per group. **j**–**l** Immunohistochemical (IHC) and immunofluorescence (IF) staining for OCN and RUNX2 of bone sections. Scale bar, 100 μm. **m** Western blot images and quantification of osteogenesis-related markers in bone tissue of mice from CTRL and HFD&STZ group. *n* = 6 per group. **n** Quantitative PCR (qPCR) analysis of osteogenesis genes. *n* = 6 per group. Data presented as mean ± SD. *P < 0.05, **P < 0.01, ***P < 0.001

A murine model of T2DM was established through a combination of high-fat-diet (HFD) feeding and low-dose streptozotocin (STZ) injection (HFD&STZ mice) (Fig. 1a).^31^ As expected, the mice in HFD&STZ group exhibited persistent increases in body weight and fasting blood glucose levels, surpassing 11.1 mmol/L of glucose compared to CTRL mice (Fig. 1b–d), confirming the successful establishment of the T2DM mouse model.

To evaluate the bone microarchitecture, micro-computed tomography (micro-CT) analysis of femurs were conducted. HFD&STZ mice displayed significant trabecular deterioration and marked bone loss (Fig. 1e). The results indicated decreased bone volume per total volume (BV/TV), bone surface per tissue volume (BS/TV), trabecular number (Tb.N), and increased trabecular spacing (Tb.Sp) in HFD&STZ mice compared to CTRL mice(Fig. 1f), signifying severe bone loss in the former.

To unravel whether the reduced bone mass in HFD&STZ mice resulted from decreased bone formation, the dual-labeled xylenol orange assay demonstrated a significant reduction in the mineralization apposition rate (MAR) in HFD&STZ mice (Fig. 1h). Similarly, Hematoxylin and Eosin (H&E) analysis revealed declined osteoblasts under diabetic conditions (Fig. 1g). Furthermore, the expression of serum procollagen type I N-terminal propeptide (PINP), a reliable bone formation indicator, was notably decreased in the HFD&STZ mice (Fig. 1i). Consistent with these findings, immunohistochemical (IHC) and immunofluorescence (IF) staining showed a concurrent suppression of osteogenic marker expressions, specifically osteocalcin (OCN) and runt-related transcription factor 2 (RUNX2) in HFD&STZ mice (Fig. 1j-l), as well as the downregulated expression of osteogenic markers *Alpl*, *Bglap*, *Ibsp*, *Spp1*, *Runx2*, *Col1a1* evidenced by quantitative PCR (qPCR) and western blot assay (Fig. 1m-n). Collectively, lower bone turnover was observed in HFD&STZ mice, primarily attributed to defects in osteogenic function.

### In vitro T2DM mimicing suppresses osteoblast differentiation and mineralization

**Fig. 2:**
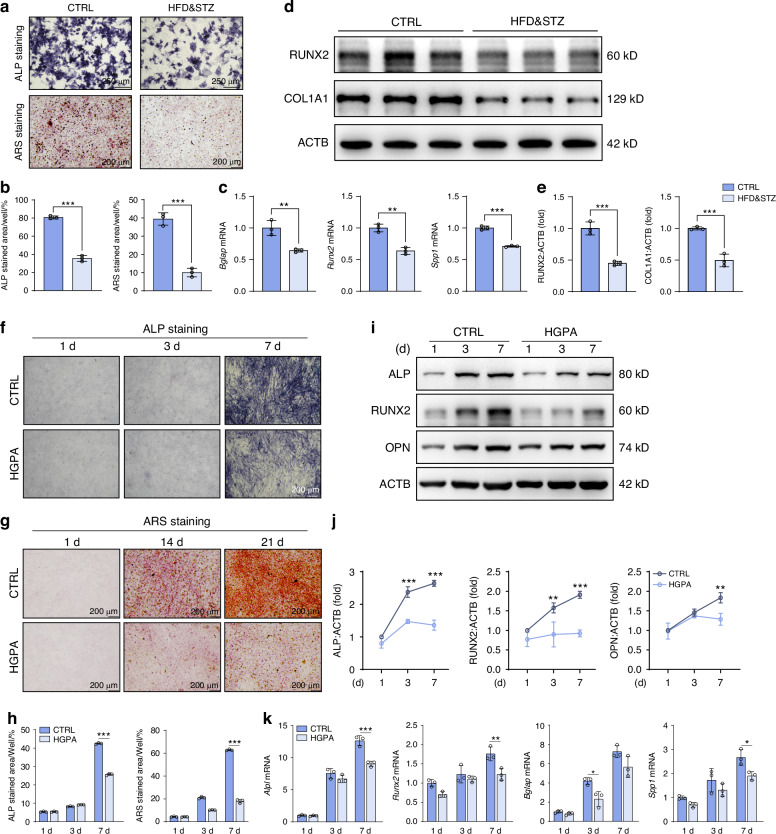
The diabetic microenvironment impairs osteoblast function in vitro. **a**, **b** Representative images and quantitative analyses of ALP (7 day) and ARS (21 day) staining of primary osteoblasts isolated from lone bones of CTRL and HFD&STZ mice in vitro. **c** Relative gene expression levels of *Bglap*, *Runx2*, *Spp1* at day 7 since induction of primary osteoblast differentiation. **d**, **e** Western blot analysis of RUNX2 and COL1A1 in primary osteoblasts. **f**–**h** Representative images and quantification of ALP and ARS staining of MC3T3-E1 cells at designated culture time points. Scale bar, 200 μm. **i**, **j** Western blot analyses of ALP, RUNX2, and OPN after cultured in osteogenic induction medium for 1-day, 3-day, and 7-day. **k** Relative gene expression levels of *Alpl*, *Runx2*, *Bglap*, and *Spp1* in MC3T3-E1 cells after induction of osteoblast differentiation. Data presented as mean ± SD. *P < 0.05, **P < 0.01, ***P < 0.001

Concurrently, we assessed the potential impact of diabetic microenvironment on osteoblasts. The results showed that the differentiation (measured by ALP staining) and mineralization (measured by ARS staining) of primary osteoblasts isolated from HFD&STZ mice were severely inhibited compared to those from the CTRL group (Fig. 2a, b). The suppressed osteogenesis was further validated by the notably decreased mRNA (*Bglap*, *Runx2*, *Spp1*) and protein (RUNX2 and COL1A1) levels (Fig. 2c–e). To emulate the diabetic milieu in vitro, we utilized 25.5 mmol/L glucose and 200 μmol/L palmitic acid (HGPA) to validate of high glucose and high fat conditions impact on osteoblast function.^4^ Following a 7-day and 21-day osteogenic induction in vitro, Alkaline Phosphatase (ALP) and Alizarin Red S (ARS) results showed the differentiation and mineralization of MC3T3-E1 cells were significantly inhibited with HGPA treatment (Fig. 2f–h). Additionally, qPCR and western blot analyses revealed a notable suppression of osteogenic markers *Alpl*, *Runx2*, *Bglap*, *Spp1* in HGPA group (Fig. 2i–k).

### Mitochondrial dysfunction and mitophagy suppression are observed in T2DM mice

**Fig. 3:**
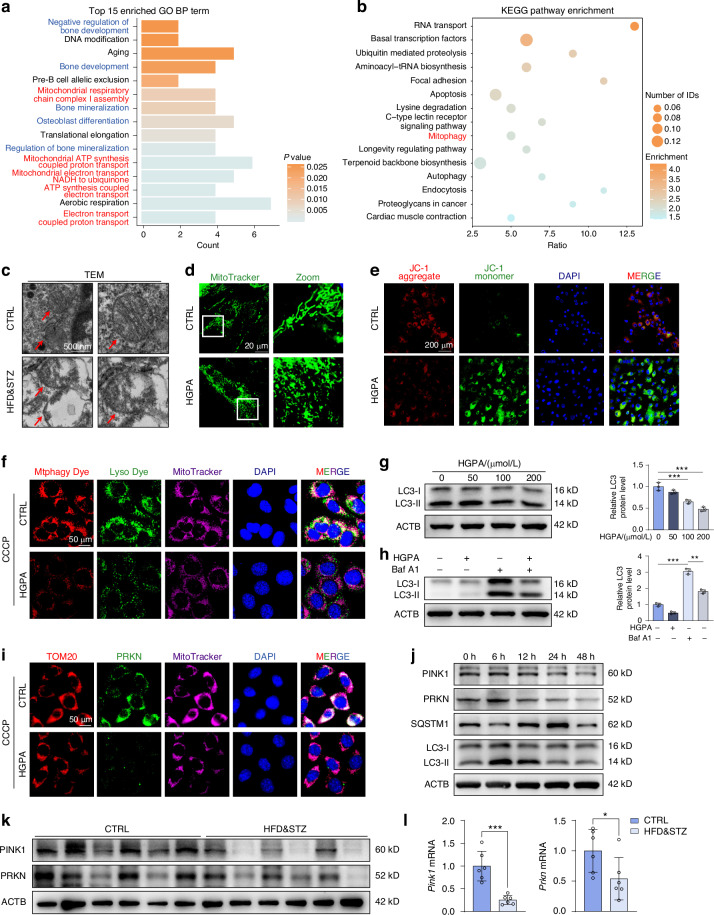
The diabetic microenvironment induces mitophagy impairment in osteoblasts. **a**, **b** Enrichment Analysis for differentially expressed genes between the CTRL and HFD&STZ groups using GO and KEGG databases. **c** Detection of osteoblast mitochondrial ultrastructure by transmission electron microscopy (TEM) in femurs from CTRL and HFD&STZ mice. **d** Visualization of mitochondria in MC3T3-E1 cells after HGPA treatment using Mitotracker Green dye. **e** Representative images of JC-1 staining in CTRL and HGPA-treated MC3T3-E1 cells. Red fluorescence represented intact mitochondria with normal membrane potential and green fluorescence represented impaired mitochondria with reduced membrane potential. Scale bar, 200 μm. **f** Representative images of MitoTracker-labeled mitochondria (deep red), Mtphagy-labeled mitophagy process (red), and Lyso Dye-labeled lysosomes (green), of which the co-localization indicates the occurrence of mitophagy. Scale bar, 50 μm. **g**, **h** Western blot images and quantification of LC3 in MC3T3-E1 cells treated with gradient concentrations of HGPA, with or without Baf-A1 (10 nmol/L). **i** Representative IF staining of TOM20 (red), PRKN (green), and MitoTracker (deep red) in MC3T3-E1 cells from CTRL and HGPA groups stimulated by CCCP for 6 h. Scale bar, 50 μm. **j** Western blot results of mitophagy-related proteins including PINK1, PRKN, SQSTM1, and LC3 in MC3T3-E1 cells stimulated by HGPA for various duration. **k** Western blot analysis of PINK1 and PRKN in bone from CTRL and HFD&STZ mice. **l** qPCR analysis of *Pink1* and *Prkn* genes. *n* = 6 per group. Data presented as mean ± SD. *P < 0.05, **P < 0.01, ***P < 0.001

In order to investigate the potential mechanisms responsible for the reduced osteogenesis in HFD&STZ mice, an RNA-seq analysis was conducted for bones of both CTRL and HFD&STZ mice. A total of 222 downregulated genes and 336 up regulated genes (*P*＜0.05, fold change ＜0.5 or >2) were identified (Fig. S1a). Subsequently, Gene Ontology (GO) and Kyoto Encyclopedia of Genes and Genomes (KEGG) enrichment analysis was performed on these differentially expressed genes, revealing a significant enrichment of mitochondrial, mitophagy and osteoblast-related processes or components (Fig. 3a, b and Fig. S1b). This finding supported the previous hypothesis of impaired osteogenesis in HFD&STZ mice.

Following this, further examination focused on mitochondrial alterations in bones. Transmission electron microscopy (TEM) was utilized to assess the ultrastructure of mitochondria, revealing prevalence of dysfunctional mitochondria in HFD&STZ mice, characterized by matrix dilation and vacuolation (Fig. 3c and Fig. S1e). Additionally, we found that HGPA-stimulated osteoblasts exhibit morphological changes in mitochondria. The results showed a decrease in elongated mitochondria and an increase in fragmented mitochondria, indicating a heightened level of mitochondrial damage (Fig. 3d and Fig. S1f). Furthermore, we employed the JC-1 fluorescent probe to assess the mitochondrial membrane potential in osteoblasts following HGPA stimulation. The results showed aggregation of green fluorescence in HGPA-stimulated osteoblasts, which indicated mitochondrial depolarization and a decline in membrane potential (Fig. 3e). Additionally, 2’,7’-dichlorodihydrofluorescein diacetate (DCFH-DA) and mitochondrial superoxide (MitoSox) staining confirmed the accumulation of ROS in osteoblasts, especially in mitochondria, upon HGPA treatment (Fig. S1c, d).

In general, dysfunctional mitochondria are typically removed through a process known as mitophagy, which is a mechanism for maintaining mitochondrial homeostasis by selectively eliminating damaged mitochondria.^32^ Hence, we postulated that the diabetic environment might hinder the mitophagy process thus impede the removal of depolarized mitochondria. To validate this hypothesis, we utilized Carbonyl Cyanide m-Chlorophenyl Hydrazine (CCCP) to induce mitophagy and monitored the mitophagy flux using dual labeling of a mitophagy-specific dye and a lysosomal dye. The results indicated that HGPA significantly inhibited mitophagy in osteoblasts (Fig. 3f). Moreover, the expression of LC3, a biomarker for autophagosomes, was significantly suppressed with HGPA treatment (Fig. 3g). Furthermore, we treated the cells with Bafilomycin A1 (Baf-A1), an inhibitor of vacuolar H+ ATPase/lysosomal acidification, to enhance autophagic flux by impeding the fusion of autophagosomes with lysosomes.^33^ Despite the reversion of autophagy due to Baf-A1, the expression of LC3 remained suppressed after HGPA treatment, suggesting that HGPA interrupted the initiation of mitophagy rather than influencing its late-stage degradation processes (Fig. 3h).

The PINK1/PRKN pathway plays a pivotal role in initiating mitophagy. Upon mitochondrial injury, PINK1 accumulates on the surface of mitochondria and promotes the recruitment of PRKN, which ubiquitinates mitochondrial membrane proteins and subsequently initiates mitophagy.^34^ Based on this, we examined whether HGPA inhibited the PINK1/PRKN pathway. As anticipated, HGPA notably decreased the co-localization of PINK1 or PRKN with mitochondria and dose-dependently reduced the expression of key mitophagy genes and proteins such as PINK1 and PRKN (Fig. 3i and Fig. S1g-j). Furthermore, we observed that mitophagy markers, including PINK1, PRKN, LC3, increased at 6 h, then declined at 24 h and 48 h after HGPA treatment (Fig. 3j). Moreover, a parallel analysis of bone tissue proteins and RNA from CTRL and HFD&STZ mice confirmed the inhibition of mitophagy activity in osteoblasts (Fig. 3k, l). In summary, these data suggested that PINK1/PRKN mediated mitophagy in osteoblasts from HFD&STZ mice or HGPA stimulation was remarkedly impaired.

### SIRT3 is down-regulated in both bones from HFD&STZ mice and osteoblasts receiving HGPA stimulation

**Fig. 4:**
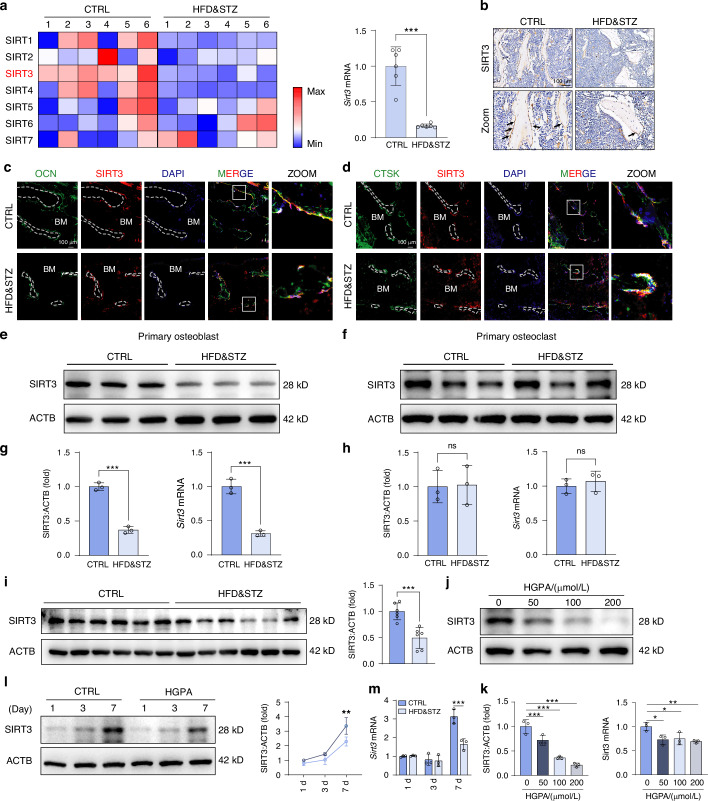
SIRT3 is reduced in HFD&STZ mice and HGPA-treated osteoblasts. **a** Heatmap demonstration of Sirtuin family (*Sirt1-7*) and expression of *Sirt3* in bone from CTRL and HFD&STZ mice. **b** IHC staining of SIRT3 in bone sections. **c**, **d** IF analysis of bone sections showing co-localization of SIRT3 with osteoblast marker (OCN) or osteoclast marker (CTSK). Scale bar, 100 μm. **e**, **f** Western blot analysis of SIRT3 in primary osteoblasts and osteoclasts from CTRL and HFD&STZ mice. **g**, **h** Quantification of western blot and qPCR analysis of *Sirt3* gene expression in osteoblasts and osteoclasts from CTRL and HFD&STZ mice. **i** Western blot analysis and quantification of SIRT3 expression in bone tissue from CTRL and HFD&STZ mice. **j**–**m** Western blot analysis and quantification of SIRT3 protein in MC3T3-E1 cells cultured for different time and concentration of HGPA. Data presented as mean ± SD. *P < 0.05, **P < 0.01, ***P < 0.001

Moving forward, we investigated the underlying mechanisms for mitophagy suppression in osteoblasts during diabetic bone loss. Given the established involvement of the sirtuin family in various biological processes, particularly in maintaining mitochondrial homeostasis, we examined the expression of sirtuin family members in CTRL and HFD&STZ mice. Changes of other members were shown in Fig. 4a, among which SIRT3 exhibited the most distinct changes, consistent with IHC staining (Fig. 4b). Notably, previous studies have indicated that during osteogenic differentiation, *Sirt3* mRNA is more differentially expressed compared to other family members.^35^ Therefore, we hypothesized that SIRT3 might participate in the mitophagy dysfunction observed in osteoblasts of HFD&STZ mice. To further confirmed that SIRT3 was specifically decreased in osteoblasts, we conducted co-staining of SIRT3 with osteoblast marker OCN or osteoclast marker Cathepsin K (CTSK) in bone sections. A notable decrease in the co-localization of SIRT3 with the osteoblast marker OCN was observed in HFD&STZ mice, while no significant alteration was noted with the osteoclast marker CTSK (Fig. 4c, d). Subsequently, primary osteoblasts and osteoclasts which was induced from bone marrow-derived monocytes, were isolated and cultured for in vitro analysis, of which the results indicated SIRT3 suppression was specific to osteoblasts (Fig. 4e–h). Moreover, analysis of both in vivo and in vitro samples further supported the downregulation of SIRT3 in T2DM mice and MC3T3-E1 cell with HGPA treatment. (Fig. 4i–k). Together, these data suggested SIRT3 was significantly decreased in osteoblasts from HFD&STZ mice or HGPA treatment.

### SIRT3 activation ameliorates HGPA-induced osteoblast dysfunction by promoting mitophagy

**Fig. 5:**
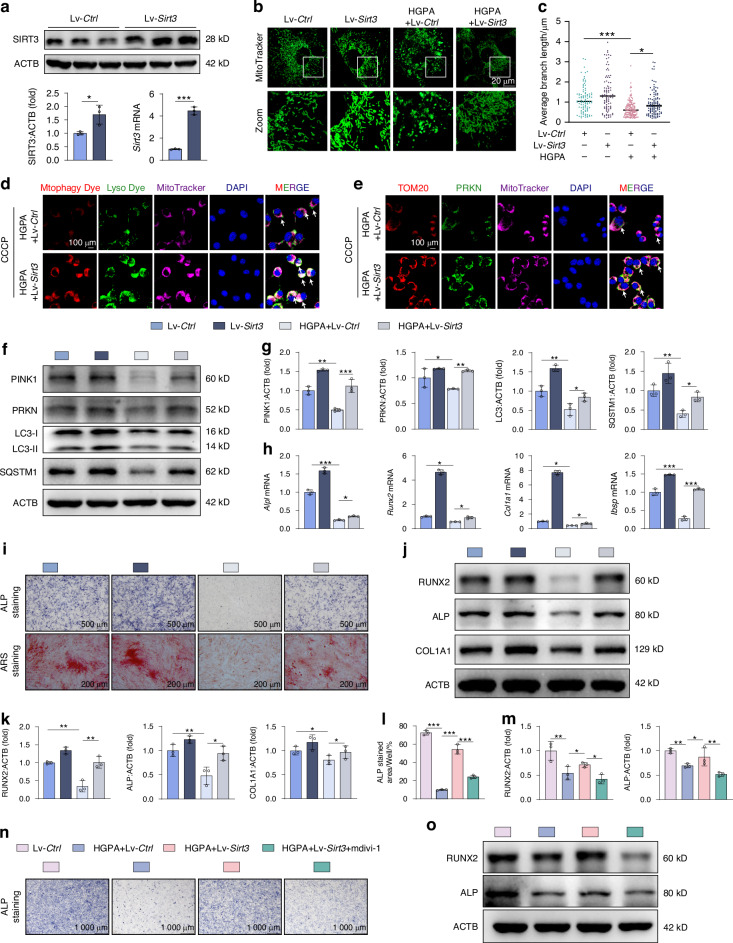
Activation of SIRT3 promotes mitophagy and ameliorates osteoblast dysfunction in MC3T3-E1 cells. **a** Relative protein and mRNA expression of SIRT3 in MC3T3-E1 cells transfected with Lv-*Ctrl* and Lv-*Sirt3*. **b**, **c** Representative images and quantification of mitochondria in Lv-*Ctrl*, Lv-*Sirt3*, HGPA+Lv-*Ctrl*, HGPA+Lv-*Sirt3* groups. Scale bar, 20 μm. **d** Representative images of MitoTracker-labeled mitochondria (deep red), Mtphagy Dye-labeled mitophagy (red), and Lyso Dye-labeled lysosomes (green), of which the co-localization indicates the occurrence of mitophagy. Scale bar, 100 μm. **e** Representative IF staining of TOM20 (red), PRKN (green), and MitoTracker (deep red) in MC3T3-E1 cells from HGPA+Lv-*Ctrl* and HGPA+Lv-*Sirt3* groups stimulated by CCCP for 6 h. Scale bar, 100 μm. **f**, **g** Western blot images and quantification of mitophagy-related proteins, including PINK1, PRKN, LC3, and SQSTM1 in the above groups. **h** qPCR analysis of *Alpl*, *Runx2*, *Col1a1* and *Ibsp* genes in 3 independent replicative experiments. **i** Representative images and quantification of ALP (7 days) and ARS (21 days) staining of MC3T3-E1 cells from above four group. **j**, **k** Western blot results and quantitative analysis of RUNX2, ALP, COL1A1 in MC3T3-E1 cells. **l**, **n** Representative images and quantification of ALP staining of MC3T3-E1 cells treated with HGPA, HGPA + HKL and HGPA+mdivi-1. Scale bar, 1 000 μm. **o**, **m** Western blot analysis of RUNX2 and ALP in MC3T3-E1 cells with different intervention. Data presented as mean ± SD. *P < 0.05, **P < 0.01, ***P < 0.001

To confirm the potential involvement of SIRT3 in treating diabetic osteoporosis through mitophagy modulation, we demonstrated that Honokiol (HKL) or lentivus overexpressing *Sirt3* (Lv-*Sirt3*) could upregulate SIRT3 in a dose-dependent manner, effectively reversing the inhibition of SIRT3 induced by HGPA (Fig. 5a and Fig. S2a-d). Ultrastructural images revealed a notable decrease in the number of damaged mitochondria with HGPA following HKL treatment (Fig. S3a). MitoTracker staining indicated that HKL-induced SIRT3 activation or Lv-*Sirt3* led to the restoration of fragmented mitochondrial morphology to a healthier state (Fig. 5b, c and Fig. S3b). DCFH-DA and MitoSox staining demonstrated that HKL alleviated the accumulation of ROS induced by HGPA in osteoblasts (Fig. S2e, f).

As previously discussed, exposure to HGPA resulted in a reduction of mitophagy in osteoblasts, with key regulators PINK1 and PRKN showing diminished mitochondrial localization. Notably, these abnormalities were significantly attenuated by SIRT3 agonist HKL or Lv-*Sirt3* treatment (Fig. 5d, e, Fig. S2g and Fig. S3c, d). Both protein and gene expressions of mitophagy factor such as PINK1 and PRKN provide compelling evidence that SIRT3 markedly mitigated the mitophagy impairment induced by HGPA and maintained mitochondrial homeostasis (Fig. 5f–h and Fig. S3e–g).

Next, we sought to investigate whether restoring mitophagy could alleviate osteoblast impairment induced by HGPA upon treatment with HKL or Lv-*Sirt3*. As anticipated, ALP and ARS staining demonstrated that HGPA-triggered inhibition of osteoblast formation and mineralization was effectively reversed by HKL or Lv-*Sirt3* (Fig. 5i and Fig. S3h–j). Remarkably, compared to osteoblasts receiving HGPA treatment, there was a significant upregulation in the expression of osteogenic markers such as ALP, RUNX2, and collagen type I alpha 1 chain (COL1A1) after HKL treatment (Fig. 5j, k and Fig. S3 k, l). To confirm the involvement of mitophagy in SIRT3-mediated mitigation of osteoblast dysfunction, we utilized mdivi-1 to inhibit mitophagy. The ALP staining results revealed that the presence of mdivi-1 significantly reserved the improvements in osteoblast formation with HGPA and HKL or Lv-*Sirt3* treatment, consistent with the expression levels of osteoblast markers including ALP and RUNX2 (Fig. 5n, o and Fig. S3m, n). Therefore, our data demonstrated that SIRT3 plays a crucial role in regulating osteoblast differentiation and mineralization processes through the modulation of mitophagy.

### SIRT3 deacetylates FOXO3 to restore *Prkn* gene transcription in diabetic osteoblasts

**Fig. 6:**
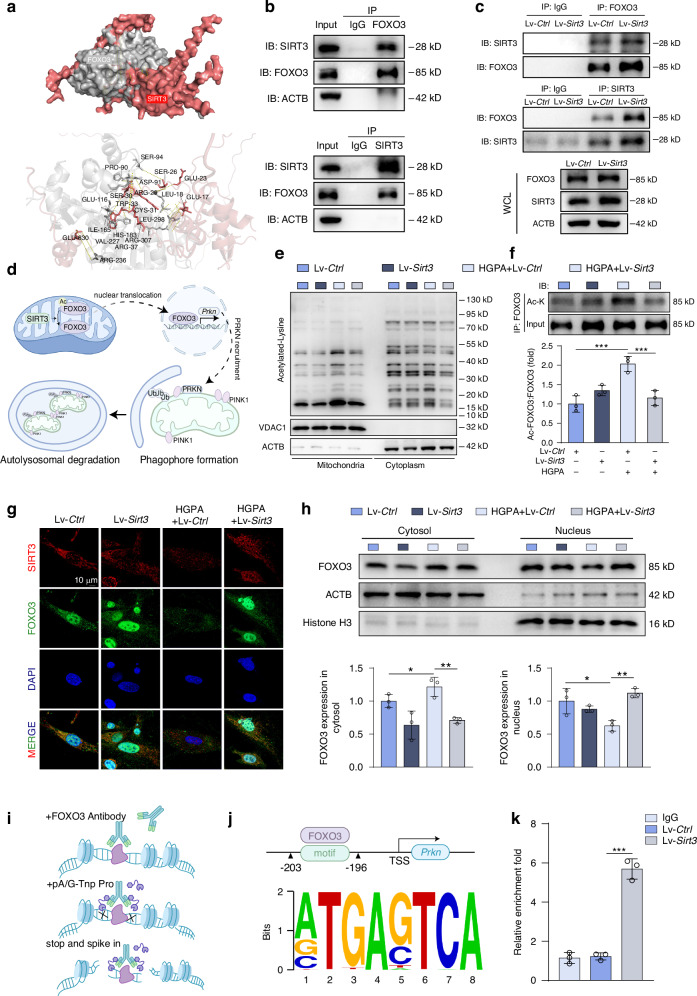
SIRT3 deacetylates FOXO3 to regulate *Prkn* gene transcription. **a** The molecular docking image of SIRT3 and FOXO3. **b**, **c** CoIP of SIRT3 and FOXO3 with or without SIRT3 transfection in MC3T3-E1 cells. **d** Schematic illustration of the mechanism that SIRT3 deacetylates FOXO3, thereby improve *Prkn*-mediated mitophagy. **e** Acetylation status of mitochondrial and cytoplasmic proteins was determined by western blot using pan-acetylated lysine antibody. Blue indicates Lv-*Ctrl*, dark blue indicates Lv-*Sirt3*, gray indicates Lv-*Ctrl* + HGPA and dark gray indicates Lv-*Sirt3* + HGPA. The same color scheme applies to (**f**, **h**). **f** Western blot analysis of acetylated FOXO3 in MC3T3-E1 cells with or without SIRT3 overexpression and HGPA stimulation. **g** Representative IF images of FOXO3 in MC3T3-E1 cells. Scale bar, 20 μm. **h** Western blot analysis of nuclear-localized and cytosolic FOXO3 in MC3T3-E1 cells. **i** Schematic representation of CUT&Tag experiment using anti-FOXO3 antibody, followed by DNA purification and qPCR analysis with primers designed for predicted binding sites (generated by Figdraw). **j** The binding motif for FOXO3 included in the promoter region of the *Prkn* gene was predicted by JASPAR. **k** CUT&Tag-qPCR assay of *Prkn* enriched by FOXO3 antibody. Data presented as mean ± SD. *P < 0.05, **P < 0.01, ***P < 0.001

FOXO3, a transcription factor crucial for cellular metabolism, mitochondrial dysfunction, and response to oxidative stress, is implicated in the pathogenesis of neurodegenerative and age-related disorders.^36,37^ We postulated that the SIRT3/FOXO3 axis may be pivotal in driving mitophagy in osteoblasts. To validate the regulatory relationship between SIRT3 and FOXO3, we conducted a molecular docking analysis which indicated that SIRT3 binds to FOXO3 with a high affinity (Fig. 6a). Furthermore, coimmunoprecipitation (CoIP) assay additionally proved that SIRT3 interacted with FOXO3 in MC3T3-E1 cells with or without SIRT3 transfection (Fig. 6b, c). Given the pivotal role of SIRT3 as a mitochondria-localized deacetylase, we proceeded to examine the acetylation of mitochondrial proteins to elucidate the regulatory mechanism of SIRT3 on mitophagy (Fig. 6d). The results demonstrated that Lv-*Sirt3* significantly reversed the hyperacetylation of mitochondrial proteins caused by HGPA (Fig. 6e). Furthermore, immunoprecipitation assay confirmed that HGPA increased the acetylation of FOXO3 in MC3T3-E1 cells, which was reversed by SIRT3 overexpression (Fig. 6f). Given that the acetylation status of FOXO3 might influence its subcellular localization, we proceeded to examine the subcellular distribution of FOXO3.^38^ The IF analysis revealed that HGPA markedly suppressed the nuclear translocation of FOXO3, while the addition of Lv-*Sirt3* or HKL reversed this phenomenon (Fig. 6g and Fig. S4a). Furthermore, western blot analysis supported these findings, showing that SIRT3 regulates FOXO3 acetylation thus promoting its nuclear translocation (Fig. 6h).

Considering its transcriptional activity, we hypothesized that FOXO3 might bind to the promoter of the *Prkn* gene to regulate its transcription. Refering to the JASPAR database (www.jaspar.genereg.net), we identified a conserved FOXO3-binding sequence in the *Prkn* promotor (Fig. 6j) and designed primers according to the predicted binding site. Prompted by this discovery, we carried out a CUT&Tag assay (Fig. 6i) to ascertain the direct interaction of FOXO3 with *Prkn*’s promoter in MC3T3-E1 cells. Our data unveiled a marked enhancement of FOXO3-promoter binding activity following Lv-*Sirt3* intervention, suggesting a direct regulatory relationship where FOXO3 modulate *Prkn*’s transcriptional activity (Fig. 6k). The reduced FOXO3 binding paralleled decreased PRKN-mediated mitophagy, while SIRT3 activation effectively reinstates this binding, thereby enhancing mitophagic activity mediated by PRKN in MC3T3-E1 cells.

### In vivo *Sirt3*-overexpression rescues HFD&STZ-induced skeletal mitophagy defects

**Fig. 7:**
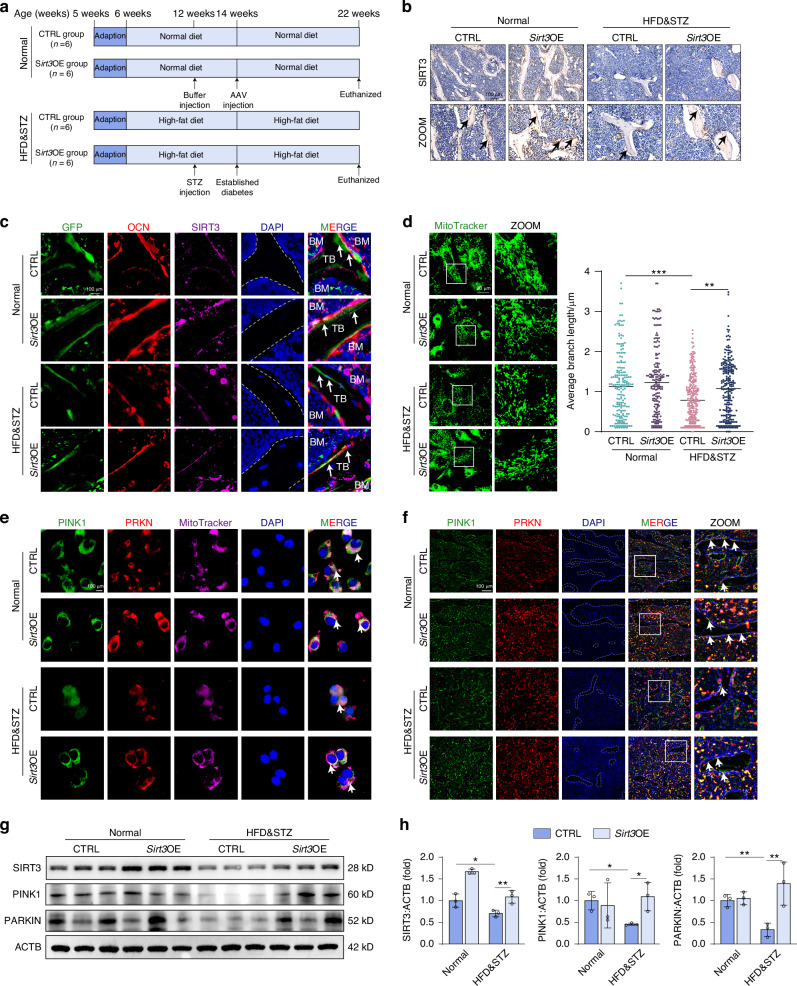
*Sirt3*-overexpression rescues HFD&STZ-induced mitophagy defects. **a** Schematic illustration of the establishment T2DM with injection of Adeno-associated virus (AAV) overexpressing *Sirt3*. **b** IHC staining for SIRT3 of bone sections in CTRL and *Sirt3*OE group from normal and HFD&STZ mice. Scale bar, 100 μm. **c** Representative IF images of bone sections showing co-localization of OCN (red), SIRT3 (deep red), and AAV-GFP (green). Scale bar, 100 μm. **d** Representative images and quantification of mitochondria stained with MitoTracker Green of primary osteoblasts from above groups. **e** The presentative IF images of PINK1 and PRKN with mitochondria in primary osteoblast from CTRL, *Sirt3*OE, HFD&STZ, and HFD&STZ+*Sirt3*OE mice. **f** The presentative IF images of PINK1 and PRKN in bone tissues. **g**, **h** Western blot images and quantification of mitophagy markers in bones from each group. Data presented as mean ± SD. *P < 0.05, **P < 0.01, ***P < 0.001

In our subsequent investigation, we intend to explore the potential of SIRT3 overexpression in bone tissue to reverse bone loss by recovering mitophagy in HFD&STZ mice. Adeno-associated virus (AAV), a nonenveloped parvovirus with high transduction efficiency and low immunogenicity, is a promising viral vector for gene therapy. To target bone tissue, a bone-targeting peptide motif (AspSerSer)6 was incorporated into the AAV-VP2 capsid protein and administered for *Sirt3*-overexpression (Fig. 7a).^39,40^ Notably, the overexpression of SIRT3 did not affect the body weight or blood glucose levels in mice (Fig. S5a). Fluorescence microscopy revealed minimal GFP expression in the liver and lungs, with predominant expression observed in osteoblasts within the bone matrix (Fig. S5b, c). The successful overexpression of SIRT3 was confirmed through various experimental techniques including IHC staining, western blot analysis, and qPCR-based gene expression assays (Fig. 7b, c and Fig. S5d).

Subsequently, we examined the mitochondrial morphology in primary osteoblasts using MitoTracker probes. Consistent with previous observations, osteoblasts from HFD&STZ mice exhibited fragmented mitochondria. However, there was a marked improvement in mitochondrial morphology following SIRT3 overexpression (Fig. 7d). Furthermore, we performed immunofluorescence staining of primary osteoblasts and tissue sections to further examine the interaction of SIRT3 and PINK1-PRKN. Consistent with the in vitro results, the expression of PINK1 and PRKN in primary osteoblasts from HFD&STZ mice was reduced. However, after treatment with AAV-*Sirt3* (Sirt3OE), the expression of PINK1 and PRKN was partially restored (Fig. 7e, f). Concurrently, the expression levels of protein PINK1 and PRKN were significantly increased compared to those in HFD&STZ mice (Fig. 7g, h and Fig. S6a). Together, these findings suggested that *Sirt3*-overexpression effectively restored mitophagy activity, thereby mitigating osteogenic mitochondrial damage in HFD&STZ mice.

### In vivo *Sirt3*-overexpression reverses HFD&STZ-induced bone loss

**Fig. 8:**
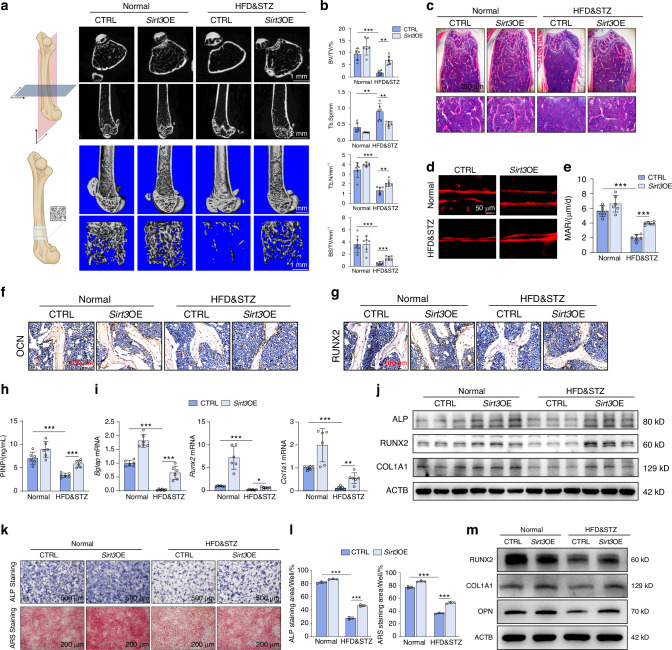
Overexpression of *Sirt3* reverses bone loss in HFD&STZ mice. **a** Representative micro-CT images of femurs from different groups of mice. **b** Quantification of bone parameters by means of micro-CT, including BV/TV, Tb.Sp, Tb.N, and BS/TV. **c** Representative images of H&E staining of bone sections from different groups. **d**, **e** High magnification and quantification of xylenol orange double labeling of the femur from different group mice. Scale bar, 50 μm. **f**, **g** IHC staining against OCN and RUNX2 of bone sections. Scale bar, 100 μm. **h** Serum concentration of PINP. *n* = 6 per group. **i** qPCR analysis of osteogenesis genes including *Bglap*, *Runx2*, *Col1a1* mRNA levels. *n* = 6 per group. **j** Western blot images and quantification including ALP, RUNX2, and COL1A1 proteins of bone tissue from different group. **k**, **l** Representative images and quantification of ALP (7 days) and ARS (21 days) staining of primary osteoblasts isolated from mice in different group. **m** Western blot analysis of RUNX2, COL1A1, and OPN proteins in primary osteoblasts from abovementioned groups. Data presented as mean ± SD. *P < 0.05, **P < 0.01, ***P < 0.001

Given the pivotal role of mitophagy in osteogenesis, we proceed to investigate whether restoration of mitophagy through SIRT3 intervention could ameliorate the impairment of bone formation in HFD&STZ mice. As expected, the reduced bone mass in the femurs of HFD&STZ mice was largely restored following AAV-*Sirt3* injection, accompanied by increased BV/TV, Tb.N, and BS/TV, as well as decreased Tb.Sp (Fig. 8a, b). Concurrently, H&E staining and dual-labeled xylenol orange experiments supported the beneficial effects of *Sirt3*-overexpression on bone formation in HFD&STZ mice (Fig. 8c–e). IHC and IF analyses of OCN and RUNX2 revealed an impairment in osteoblastogenesis in T2DM mice. However, following treatment with AAV-*Sirt3*, a significant increase was observed in the number of osteoblasts positively stained for both OCN and RUNX2 markers (Fig. 8f, g and Fig. S7a). These findings align with serological data demonstrat that SIRT3 therapy elevated PINP expression in the serum of HFD&STZ mice (Fig. 8h). Collectively, those results suggested that AAV- *Sirt3* treatment significantly recovered osteogenesis and improved bone health in HFD&STZ mice.

Meanwhile, qPCR assays demonstrated that decreased expression of osteogenic markers such as *Bglap*, *Runx2*, *Col1a1* in bones of HFD&STZ mice was recovered after *Sirt3*-overexpression, as well as protein ALP, RUNX2, and COL1A1 (Fig. 8i, j and Fig. S7b). Moreover, primary osteoblasts isolated from the mice showed that SIRT3 positively influenced osteoblast differentiation and mineralization, as confirmed by ALP and ARS staining, which was further corroborated the protein level (RUNX2, COL1A1, and OPN) (Fig. 8k–m and Fig. S7c). Taken together, these results demonstrate that overexpression of *Sirt3* using bone-targeted virus can mitigate bone loss induced by T2DM via upregulating the PINK1/PRKN mediated mitophagy.

## Discussion

This study demonstrates that SIRT3 is down-regulated in osteoblasts during the progression of T2DM, which partially contributes to impaired bone formation by hindering PINK1/PRKN mediated mitophagy. Restoration of SIRT3 expression or activity enhances mitophagy thus significantly alleviates bone loss of T2DM. Mechanistically, we show that SIRT3 reverses excessive acetylation of FOXO3 thus recovered mitophagy process by transcriptionally upregulating *Prkn*. Collectively, these findings provide new insights into the regulation of osteoblast homeostasis under T2DM circumstances and processes convincing potential for clinical translation.

With the aging of the global population, the prevalence of diabetes and osteoporosis is becoming increasingly threatening.^41^ Over 90 000 osteoporotic fractures occur globally each year, constituting a substantial challenge to both human health and social economics.^42^ Among the etiological factors of osteoporotic fractures, we note that T2DM stands out as a significant contributing factor. Diabetic microenvironment, marked by hyperglycemia and insulin resistance, causes the excessive accumulation of pathogenic metabolites in bone tissue.^43^ Consistent with previous research findings, we observed reduced bone mass characterized by impaired bone formation in T2DM mice (Fig. 1). For in vitro study, we utilized high glucose and palmitic acid (HGPA) to mimic diabetic environment and the results showed osteogenic differentiation was significantly inhibited (Fig. 2). Nevertheless, the mechanisms underlying osteoblast dysfunction in this diabetic microenvironment remain to be elucidated.

Mitochondria, energy center of cells, play a crucial role in maintaining cellular functions and overall metabolic balance, which has been reported to be closely related to T2DM progression.^44^ Previous studies showed that high glucose conditions severely impair mitochondrial functions of osteoblasts, exhibiting as reduction in mitochondrial cristae and accumulation of ROS.^45^ Similarly, our RNA-seq and TEM analysis demonstrated that mitochondria was markedly damaged in T2DM mice. And our in vitro study showed there was a disruption in mitochondrial membrane potential, indicating the accumulation of damaged mitochondria in osteoblasts with HGPA treatment (Fig. 3).

Selective autophagic clearance of dysfunctional mitochondria, termed mitophagy, represents a pivotal mechanism in intracellular mitochondrial quality control.^46^ The PINK1/PRKN pathway has been identified as one of the most classical, pivotal, and the most extensively studied mechanisms governing the mitophagy process.^47^ Upon mitochondrial membrane depolarization, PINK1 phosphorylates ubiquitin to activate PRKN, which conjugates ubiquitin chains to mitochondrial outer membrane proteins, driving recruitment of autophagy receptors to initiate mitophagy.^48^ The dysfunction of PINK1/PRKN-mediated mitophagy has been reported to initiate various diseases such as diabetic nephropathy and cancer.^49–51^ Similarly, our research demonstrated that PINK1/PRKN-mediated mitophagy was significantly impaired in osteoblasts upon HGPA treatment (Fig. 3).

SIRT3, an NAD^+^-dependent deacetylase primarily localized in mitochondria, plays a pivotal role in various biological processes such as cellular metabolism, stress response, cell cycle, and mitophagy.^52^ Therefore, we hypothesized that restoring SIRT3 expression may alleviate bone loss in T2DM mice via recuperating PINK1/PRKN-mediated mitophagy. Through a series of in vitro and in vivo experiments, we explored the potential role of SIRT3 in diabetic osteoporosis and its impact on mitophagy of osteoblast. As expected, decreased SIRT3 expression accompanied with impaired mitophagy were observed in T2DM (Fig. 4). And the dysfunction of osteoblast differentiation and mitophagy was recovered with an SIRT3 agonist HKL treatment (Fig. 5). Moreover, restoring SIRT3 expression using AAV-delivered systems enhances the mitophagy and improves bone formation in T2DM mice (Fig. 7 and Fig. 8). Meanwhile, the enhanced differentiation and mineralization of osteoblasts after SIRT3 intervention was reversed by the mitophagy inhibitor mdivi-1 (Fig. 5). These data revealed that SIRT3 attenuated dysfunction of bone formation in T2DM mice via promoting PINK1/PRKN-mediated mitophagy.

As a deacetylase, SIRT3 maintains cellular homeostasis via modulating acetylation of certain proteins.^53,54^ In our experiments, the acetylation status of mitochondria proteins was abnormally elevated upon HGPA stimulation, along with the downregulation of SIRT3 in diabetic microenvironment. In a previous study, downregulated SIRT3 expression led to suppression of the FOXO3/PINK1/PRKN signaling cascade, hindering mitophagy and consequently resulting in neuron apoptosis.^55^ Align with the previous study, *Sirt3* overexpression significantly inhibited the hyper-acetylation of the transcription factor FOXO3, facilitating its nuclear translocation (Fig. 6). JASPAR analysis revealed a potential FOXO3 binding motif in the *Prkn* promoter and our CUT&Tag experiment confirmed increased FOXO3 recruitment to the *Prkn* promoter post-SIRT3 activation, indicating restored transcriptional regulation of *Prkn* by FOXO3 upon SIRT3 restoration. (Fig. 6). In conclusion, our data suggests a restorative effect on the FOXO3-mediated control of *Prkn* expression, regulated through SIRT3-dependent deacetylation, which is pivotal in mitochondrial quality control processes during diabetic osteoporosis.

Nonetheless, our study also entails several aspects that warrant further in-depth exploration. Our hypotheses were validated in preclinical models, the clinical application and effectiveness in actual T2DM patients warrant further investigations. Furthermore, a deeper understanding is required regarding the mechanisms driving the downregulation of SIRT3 in the context of diabetic osteoporosis, as well as its distinct functions across different diabetes subtypes, highlighting the need for meticulous further research. Additionally, further attention should be given to changes in cortical bone under conditions of diabetes. Based on this study, the development of therapies that specifically boost SIRT3 activity in osteoblasts can be pursued.

**Scheme 1:**
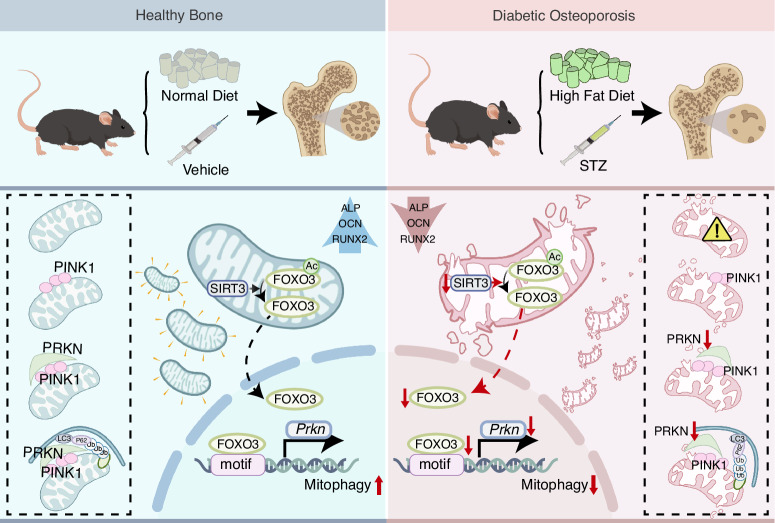
Schematic illustration of the mechanism that SIRT3 activation effectively attenuates the impairment of osteoblast formation and bone loss in T2DM by revitalizing the PINK1/PRKN-mediated mitophagy

In summary, our study provides the first confirmation of the pivotal role of SIRT3 in diabetic osteoporosis. The results demonstrated that SIRT3 activation effectively attenuates the impairment of osteoblast formation and bone loss in T2DM by revitalizing the PINK1/PRKN-mediated mitophagy. Mechanically, SIRT3 regulated the deacetylation and nuclear translocation of FOXO3, and enhanced recruitment of FOXO3 to the promoter of *Prkn* gene. Taken together, our investigation identifies SIRT3/FOXO3 as a critical regulatory cascade in osteoblasts, suggesting a potential novel therapeutical strategy for diabetic osteoporosis (Scheme 1).

## Materials and methods

### Animal experiments

All animal experiment conducted in this study were complied with the guidelines of the Committee on the Use and Care of Animal of the Nanjing Drum Tower Hospital, Nanjing University Medical School (Nanjing, China). Five-week-old mice were purchased from GemPharmatech Co., Ltd and housed in individually ventilated cages under specific pathogen-free conditions. The mice were provided sterile feed and maintained under a 12 h light/dark cycle, with the room temperature controlled at 24 ± 2 °C and relative humidity regulated within the range of (55 ± 5)%.

To establish a mouse model for human T2DM, 6-week-old C57BL/6 J male mice were randomly divided into two groups after a one-week acclimatization period: a CTRL group (CTRL, *n* = 6) and a T2DM group (HFD&STZ, *n* = 6). To assess the potential therapeutic effect of *Sirt3*-overexpression on osteoporosis in diabetic mice, the animals were further divided into four groups: Ctrl group (*n* = 6), Ctrl+AAV-*Sirt3* group (*n* = 6), HFD&STZ group (*n* = 6), HFD&STZ + AAV-*Sirt3* group (*n* = 6). Mice in the Ctrl and Ctrl+ AAV-*Sirt3* groups were given standard feed (D12450B, Research Diet) and received a citrate buffer (HY-B1610N, MedChemExpress, Monmouth Junction, NJ, USA) injection, while those in the HFD&STZ and HFD&STZ + AAV-*Sirt3* groups were fed a high-fat diet (HFD, D12492, Research Diet) for 6 weeks followed by intraperitoneal injections of 35 mg/kg STZ (Cat No.60256ES80; Yeasen, Shanghai, China) diluted using citrate buffer once daily for 3 consecutive days. Subsequently, mice in the HFD&STZ + AAV-*Sirt3* and Ctrl+AAV-*Sirt3* groups were administered with AAV9-delivered SIRT3 (Life-iLab, Shanghai, China), while the remaining two groups of mice received injections of a control virus (AAV-*ctrl*). Body weight and fasting blood glucose levels were measured weekly or biweekly. Only mice with fasting blood glucose levels exceeding 11.1 mmol/L, as measured from a tail blood sample, were verified as diabetic for further investigation. All mice were euthanized 10 weeks post-STZ injection for subsequent histological and biochemical analyses.

### Cell culture

The osteoblastic cell line MC3T3-E1 (IM-M080) was purchased from IMMOCELL (Xiamen, Fujian, China) and cultured in α-MEM (A19511, HAKATA) supplemented with 10% FBS (BC-SE-FBS06C, BioChannel Biological Technology Co., Ltd) and 1% Penicillin-Streptomycin Solution (PB180120, Pricella Life Science&Technology Co., Ltd). Cell cultures were either passaged using trypsin-EDTA solution (H0516, HAKATA) or cryopreserved with a serum-free cell freezing medium (CSP042, ZQXZ-bio) with cryovial (606802, NEST Biotechnology). To mimic an in vivo hyperglycemic and hyperlipidemic condition, 200 μmol/L palmitic acid (PA, Kunchuang Technology, Xi’an, Shaanxi, China) and 25.5 mmol/L glucose (G7021, Merck) were supplemented. Upon reaching around (70–80)% confluence in culture medium, cells were replaced induction medium to stimulate osteoblast differentiation and added with or without HGPA. The induction medium was replaced every two days along with the addition of HGPA. Honokiol (10 μmol/L, HKL, T3001) was purchased from TargetMol (USA) to serve as the agonist of SIRT3. Additionally, mdivi-1 (10 μmol/L, HY-15886), Bafilomycin A1 (10 nm, Baf-A1), CCCP (10 μmol/L, HY-100941), dexamethasone (HY-14648), β-Glycerophosphate disodium salt pentahydrate (HY-D0886), and vitamin C (HY-B0166) were purchased from MedChemExpress (Monmouth Junction, NJ, USA).

### Isolation of primary osteoblasts

For mice receiving various interventions, the bilateral hindlimbs were collected and meticulously cleaned, removing the adjacent muscle and adipose tissue. Subsequently, the limbs were finely chopped using scissors and washed thrice with phosphate-buffered saline (PBS, SP02020500, Sperikon Life Science & Biotechnology co.,ltd). Following this, the tissue was immersed in a shaking incubator at 37 °C for 2 h in diluted type I collagenase (C917425, Macklin). The digested tissue was then suspended in complete culture medium (α-MEM) supplemented with 10% FBS. Upon reaching approximately (70–80)% confluence, cells at passage 2 were employed for subsequent experiments. The osteogenic induction process is the same as that for MC3T3-E1 cells.

### Stable cell line generation

*Sirt3* lentiviral vectors were purchased from PackGene Biotech. A preliminary experiment was conducted in accordance with the manufacturer’s instructions to determine the optimal multiplicity of infection (MOI) for Lv-*Sirt3* transduction. Subsequently, 8 × 10^4^ cells per well were seeded in a 12-well plate. On the following day, the appropriate volume of virus at the optimized MOI was added to the medium containing 5 μg/mL polybrene (HY-112735, MedChemExpress, Monmouth Junction, NJ, USA). The medium was replaced after 24 h and incubated for an additional 48 hours. Puromycin (5 μg/mL, HY-B1743A, MedChemExpress) was utilized to screen and establish stable transformants.

### Osteogenic differentiation and mineralization

Cells were seeded into 12-well plates (Jet Biofil) at a density of 2×10^5^ cells per well (counted with Countstar, IE1000, China) and cultured in osteogenic medium containing 10% FBS, 10 nmol/L dexamethasone, 10 mmol/L β-Glycerophosphate disodium salt pentahydrate and 50 μg/mL vitamin C. Osteogenic differentiation and mineralization were evaluated on day 14 and 21 using the BCIP/NBT Alkaline Phosphatase Color Development Kit (C3206, Beyotime) and Alizarin Red S Solution (ALIR-10001, cyagen). HGPA is continuously maintained in the induction medium to mimic the chronic progression of diabetes.

### Osteoclast differentiation

The primary osteoclast was induced from bone marrow-derived monocytes with the presence of Receptor Activator of Nuclear Factor kappa-B Ligand (RANKL, 50343-M07H, Sino Biological Inc). Bone marrow was flushed out after removing the muscles surrounding femurs in CTRL and HFD&STZ mice. Subsequently, the bone marrow was cultured in medium (L1051, BDBIO HangZhou China) containing 25 ng/mL M-CSF (CK02, Novoprotein, Shanghai, China) for 3 days. The cells were resuspneded using a cell scraper (CSC011025, Jet Biofil) and then plated. Afterward, 50 ng/mL RANKL was introduced to induce osteoclast differentiation. The culture medium was changed every two days until mature multinucleated osteoclasts formation.

### Micro-CT analysis

Mouse femurs were collected and fixed in 4% paraformaldehyde (PFA, HY-Y0333, MedChemExpress) for scanning as previously described in ref. ^56^. Microscopic computed tomography (micro-CT) was used to evaluate bone mass using a micro-CT scanner (VivaCT80; Scanco Medical AG, Swizerland) at a voxel resolution of 15.6 μm. The ratio of bone volume to total volume (BV/TV), the number of trabecular bones (Tb.N), bone surface per tissue volume (BS/TV), and trabecular spacing (Tb.Sp) were performed to analysis bone morphology.

### Hematoxylin and Eosin (H&E) and Immunohistochemical (IHC) staining

Femur samples were fixed with 4% PFA, decalcified for 28 days using EDTA (HY-Y0682, MedChemExpress), followed by dehydration and embedded in paraffin. Then embedded specimens were sectioned at a thickness of 5 μm and stained with hematoxylin and eosin (H&E, G4520, Beijing Solarbio Science & Technology Co., Ltd.) after deparaffinized and rehydrated. For IHC staining, deparaffinized and rehydrated sections were antigen-retrieved and incubated with 3% hydrogen peroxide. After primary antibody incubation, the slides were exposed to an HRP-conjugated secondary antibody (abs957, absin) and stained with 3,3’-diaminobenzidine at room temperature. The primary antibodies used were against OCN (bs0470R, Bioss USA), RUNX2 (R25634, Zen-bio), PINK1 (23274-1-AP, proteintech), PRKN (14060-1-AP, proteintech), and SIRT3 (HA722251, HUABIO).

### In vivo bone formation analysis

For in vivo bone formation analysis, xylenol orange (30 mg/kg, HY-W110883, MedChemExpress, Monmouth Junction, NJ, USA) was injected on the 8th day and 2th day prior to sacrifice. Following fixation and dehydration, the femurs were embedded in polymethyl methacrylate resin. The sections at a thickness of 5 μm were generated with a microtome for hard tissue and the images were captured with Olympus FV3000 laser confocal microscope. Bone dynamic histomorphometric assessment was performed utilizing Image J software.

### Immunofluorescence (IF) staining

MC3T3-E1 cells were cultured on glass bottom culture dishes (801002, NEST Biotechnology) and subjected to corresponding treatments. The cells were fixed by 4% PFA for 10 minutes and incubated with 0.1% Triton X-100 (E-IR-R122, Elabscience Biotechnology Co., Ltd). Subsequently, cells were blocked with goat serum (E-IR-R110, Elabscience Biotechnology Co., Ltd) at room temperature for 60 min, followed by overnight incubation with primary antibodies at 4 °C. The next day, secondary antibodies conjugated with Alexa Fluor 488 or Alexa Fluor 594 (A-11094 and 331594, Invitrogen) were utilized, and the cell nuclei were stained with DAPI solution (HY-K1048, MedChemExpress).

For femur sections, the same procedure was applied as described for IHC staining. Finally, images were acquired using Olympus FV3000 laser confocal microscope or Leica DMi8 THUNDER Imaging Systems. The primary antibodies used for IF staining were as follows: OCN (bs0470R, Bioss USA), RUNX2 (R25634, Zen-bio), TOM20 (A19403, ABclonal, China), PRKN (66674-1, Proteintech), PINK1 (A7131, ABclonal, China), SIRT3 (HA722251, HUABIO), CTSK (bs-1611R, Bioss), FOXO3 (66428-1, Proteintech).

### Western blot analysis

Following specific treatment, cell samples were lysed in RIPA lysis buffer (PL102, Beijing Genesand Biotech Co., Ltd) supplemented with phosphatase inhibitor cocktail (C0104, Beijing LABLEAD Inc), protease inhibitor cocktail (DI111; TransGen Biotech, China), and PMSF (HY-B0496, MedChemExpress) for 30 min at 4 °C. Bone samples were homogenated in liquid nitrogen post muscle removal, and then mixed with lysis buffer. Subsequently, the lysates underwent centrifugation at 12 000 g for 10 min using a high-speed microcentrifuge (D3024, DLAB Scientific Co. Ltd) to obtain protein. Mitochondrial protein from MC3T3-E1 cells was isolated with a Mitochondrial Isolation Kit (K1138, APExBIO, Houston, USA). Nuclear and cytoplasmic proteins were extracted using Nuclear and Cytoplasmic Protein Extraction Kit (P0027, Beyotime). Protein concentrations were quantitatively determined according to the instructions provided with the BCA Protein Assay kit (PA002, Novoprotein, Shanghai, China). Then SDS-PAGE Sample Loading Buffer (452343, Sperikon Life Science & Biotechnology Co., ltd) were added and heated at 100 °C for denaturation.

The protein samples were separated by SDS-PAGE (M00666, GenScript Corporation), and transferred to PVDF membranes (Cobetter). These membranes were blocked using quick blocking buffer (P30500, New Cell & Molecular Biotech, China) for 15 min and incubated with primary antibodies overnight, followed by the secondary antibody conjugated with horseradish peroxidase. Western blotting bands were detected with enhanced chemiluminescence kit (A508, BDBIO HangZhou China). The primary antibodies used were as follows: ALP (A0514, ABclonal, China), RUNX2 (R25634, Zen-bio), COL1A1 (A1352, ABclonal, China), OPN (FNab06033, FineTest), LC3 (ET1701-65, HUABIO), PRKN (66674-1, Proteintech), PINK1 (A7131, ABclonal, China), SQSTM1 (HA721171, HUABIO), SIRT3 (HA722251, HUABIO), Anti-Acetyllysine Rabbit mAb (PTM-105RM, PTM BIO), FOXO3 (66428-1, Proteintech), ACTB (AC026, ABclonal, China), Histone H3 (68345-1, Proteintech) and VDAC1 (41776-1, Signalway Antibody).

### RNA extraction and qPCR analysis

RNA samples were extracted utilizing a cell total RNA isolation kit (AN51L518, Life-iLab, China). For bone samples, RNA extraction was carried out using the standard Trizol reagent (R1000, LABLEAD Inc.). Briefly, surrounding muscles were removed from fresh bones and homogenized using liquid nitrogen. Then the samples resuspended in Trizol reagent and lysed for 30 min. Following this, chloroform was added to facilitate phase separation and the mixture was subjected to high-speed centrifugation at 12 000 *g*. After precipitation with isopropanol, the RNA pellet was washed with 75% ethanol and dissolved in DEPC (JDW0101, BiOligo Biotechnology Shanghai). Subsequently, cDNA synthesis was performed using the Evo M-MLV RT kit for qPCR (AG11707, ACCURATE BIOTECHNOLOGY(HUNAN) CO.,LTD, ChangSha, China). Gene expression level relative to *Actb* was determined using gene-specific primers (Generay Biotechnology, Shanghai, China) and SYBR Green (B110032, Sangon Biotech). The primer sequences used were detailed in Table S1.

### RNA sequencing

Transcriptome sequencing was conducted for bone RNA samples obtained from the CTRL and HFD&STZ mice. Each group consisted of three independent biological replicates. The total RNA was extracted and sequenced utilizing an Illumina Novaseq platform by Seqhealth Technology Co., LTD (Wuhan, China). Subsequently, gene ontology (GO) and Kyoto Encyclopedia of Genes and Genomes (KEGG) analysis were performed online in BioLadder (BioLadder.cn), with a significance level of *P* < 0.05 indicating a statistically significant difference.

### Transmission electron microscopy

Fresh bone or cell samples were fixed in 2.5% glutaraldehyde (sigma-Aldrich, G5882) at 4 °C for 24 h, followed by three washes with PBS. The specimens were then post-fixed with 1% osmium tetroxide, dehydrated, and embedded. Subsequently, the samples were sectioned into 70–90 nm utilizing a LEICA EM UC7 ultramicrotome. These sections were stained with a combination of lead citrate and uranyl acetate. Finally, the mitochondrial ultrastructural changes were visualized using a HITACHI HT7700 transmission electron microscope.

### ROS measurements

Intracellular and mitochondrial superoxide (MitoSox) were measured with CheKintonge™ reactive oxygen species (ROS) detection fluorometric assay Kit (KTB1910, Abbkine) and MitoSOX Red Mitochondrial Superoxide Indicator dye (M36009, Invitrogen™). Briefly, MC3T3-E1 cells were cultured in chamber slides (1092000, SAINING Biotechnology) and treated with or without HGPA. Then, cells were incubated with 10 μmol/L fluorescent probes for 30 minutes at 37 °C. Then the nuclei of cells were stained with DAPI. The fluorescence of the cells was visualized using Leica DMi8 THUNDER Imaging Systems.

### Enzyme-Linked Immunosorbent Assay (ELISA)

Blood samples were obtained and subsequently centrifuged at 3 000 rpm for 15 min to isolate serum. The N-Propeptide of Type I Procollagen (PINP) concentration in the serum was quantified using the ELISA kit (JL20174) obtained from Jianglai biology (Shanghai, China) according to the manufacturer’s instructions. Absorbance was measured using the Varioskan LUX multifunctional microplate reader (Thermo Fisher Scientific^TM^).

### MitoTracker staining assay and Mitochondrial membrane potential detection

Cells were seeded onto chamber slides (1092000, SAINING Biotechnology) and treated with HGPA for 48 hours. Mitochondrial morphology and mitochondrial membrane potential were assessed using Mito-Tracker Green (KGE2704, Keygen BioTECH) and Mitochondrial Membrane Potential Assay Kit (AKOP013-1, Beijing Boxbio Science & Technology Co., Ltd). According to the manufacturer’s instructions, cells were incubated with 100 nmol/L probes for 30 min at 37 °C in the dark. Subsequently, the cells were rinsed with balanced salt solution and stained with DAPI. The images were captured using an Olympus FV3000 laser confocal microscope and analyzed by Image J.

### Co-immunoprecipitation and molecular docking

After cell lysis using immunoprecipitation (IP) lysis buffer (W6001, US EVERBRIGHT, Suzhou, China), the cell lysates were incubated at 4 °C overnight with an FOXO3 antibody (66428-1, Proteintech) or SIRT3 antibody (HA722251, HUABIO) for antigen-antibody interactions. Subsequently, Protein A/G magnetic beads (HY-K0202, MedChemExpress, Monmouth Junction, NJ, USA) were introduced into the antigen-antibody mixture and incubated at room temperature for 30 min to facilitate the formation of antigen-antibody-bead complexes. Following thorough washing to eliminate non-specifically bound proteins, the beads were separated, and the supernatant containing the immunoprecipitated complexes was carefully collected for analysis via SDS-PAGE, allowing for the evaluation of protein acetylation status using pan-acetyl lysine antibody (PTM-105RM, PTM BIO), FOXO3 antibody (66428-1, Proteintech), and SIRT3 antibody (HA722251, HUABIO). To validate the regulatory relationship between SIRT3 and FOXO3, we acquired the protein structural data from AlphaFold DB (created with the AlphaFold Monomer v2.0 pipeline) and performed molecular docking using the GRAMM Web Server platform. The results were then visualized using PyMOL.

### Cleavage Under Targets and Tagmentation (CUT&Tag) assay

The CUT&Tag assay was conducted utilizing Hyperactive Universal CUT&Tag Assay Kit for Illumina Pro (TD904, Vazyme Biotech Co., Ltd). Briefly, cells were harvested and incubated with ConA Beads Pro at room temperature for 10 minutes. Afterwards, primary antibody against FOXO3 were added and allowed to incubate overnight at 4°C. The next day, pA/G-Tnp Pro reagent was employed to facilitate the transposon integration with the cell-beads complexes, thereby enhancing capture efficiency. The quantitative analysis of the captured DNA fragments was carried out using real-time quantitative polymerase chain reaction (qPCR). The primer sequences used were detailed in Table S2.

### Statistical analysis

All quantitative data are presented as the mean ± SD values of at least three separate experiments. Graphpad Prism 9 software was used for Student’s test to compare two groups or one-way ANOVA with Tukey’s post hoc test to compare among more than two groups. Significance levels *P*-value < 0.05 was considered as relatively significant levels.

## Supplementary information

Enhanced SIRT3 Expression Restores Mitochondrial Quality Control Mechanism to Reverse Osteogenic Impairment in Type 2 Diabetes Mellitus

## Data Availability

The RNA-seq data generated and analyzed during the current study are not publicly available but are available from the corresponding author on reasonable request.
