# Supplementary material for: Enhanced SIRT3 expression restores mitochondrial quality control mechanism to reverse osteogenic impairment in type 2 diabetes mellitus
Source: Bone Res. 2025 Mar 3;13:30. doi: 10.1038/s41413-024-00399-5 (PMC11873136; doi:10.1038/s41413-024-00399-5)
Supplement: Supplementary file 1 — Enhanced SIRT3 Expression Restores Mitochondrial Quality Control Mechanism to Reverse Osteogenic Impairment in Type 2 Diabetes Mellitus [file 41413_2024_399_MOESM1_ESM.docx]

Supplement Information

**Enhanced SIRT3 Expression Restores Mitochondrial Quality Control Mechanism to Reverse Osteogenic Impairment in Type 2 Diabetes Mellitus**

Yansi Xian, Bin Liu, Tao Shen, Lin Yang, Rui Peng, Hongdou Shen, Xueying An, Yutian Wang, Yu Ben, Qing Jiang*, Baosheng Guo*

**
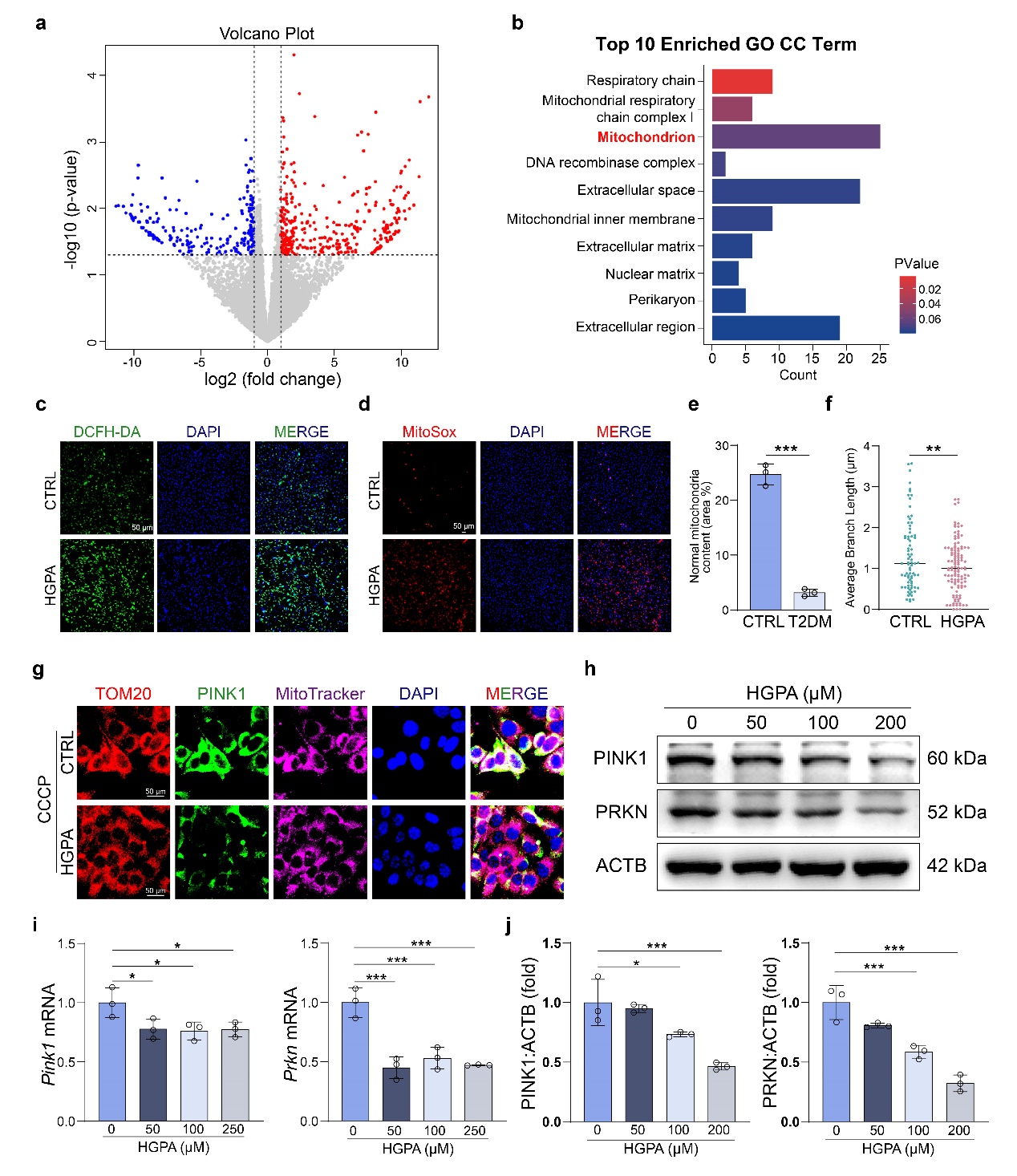
**

**Fig. S1** HGPA treatment induces mitochondrial dysfunction and suppresses mitophagy. (a) Volcano plot illustrating significantly downregulated or upregulated DEGs (Fold change ≥2) between CTRL and T2DM groups. (b) Gene Ontology (GO) enrichment analysis for downregulated genes between the CTRL and HFD&STZ groups. (c) The fluorescence images of intracellular reactive oxygen species (ROS) generation using 2’,7’-dichlorodihydrofluorescein diacetate (DCFH-DA) probe. Scale bar, 50 μm. (d) The fluorescence images of mitochondrial superoxide levels using MitoSox probe. Scale bar, 50 μm. (e) The quantification of mitochondria content in TEM. (f) The quantification of mitotracker. (g) Representative IF staining of TOM20 (red), PINK1 (green), and MitoTracker (deep red) in MC3T3-E1 cells from CTRL and HGPA groups stimulated by CCCP for 6h. Scale bar, 50 μm. (h, j) Representative western blot and quantification of mitophagy markers in MC3T3-E1 cells treated with different concentration of HGPA. (i) Transcription levels of mitophagy genes including *Pink1*, *Prkn* in MC3T3-E1 cells treated with different concentration of HGPA (50, 100, and 250 μM). Data presented as mean ± SD. **p*＜0.05, ***p*＜0.01, ****p*＜0.001.

**
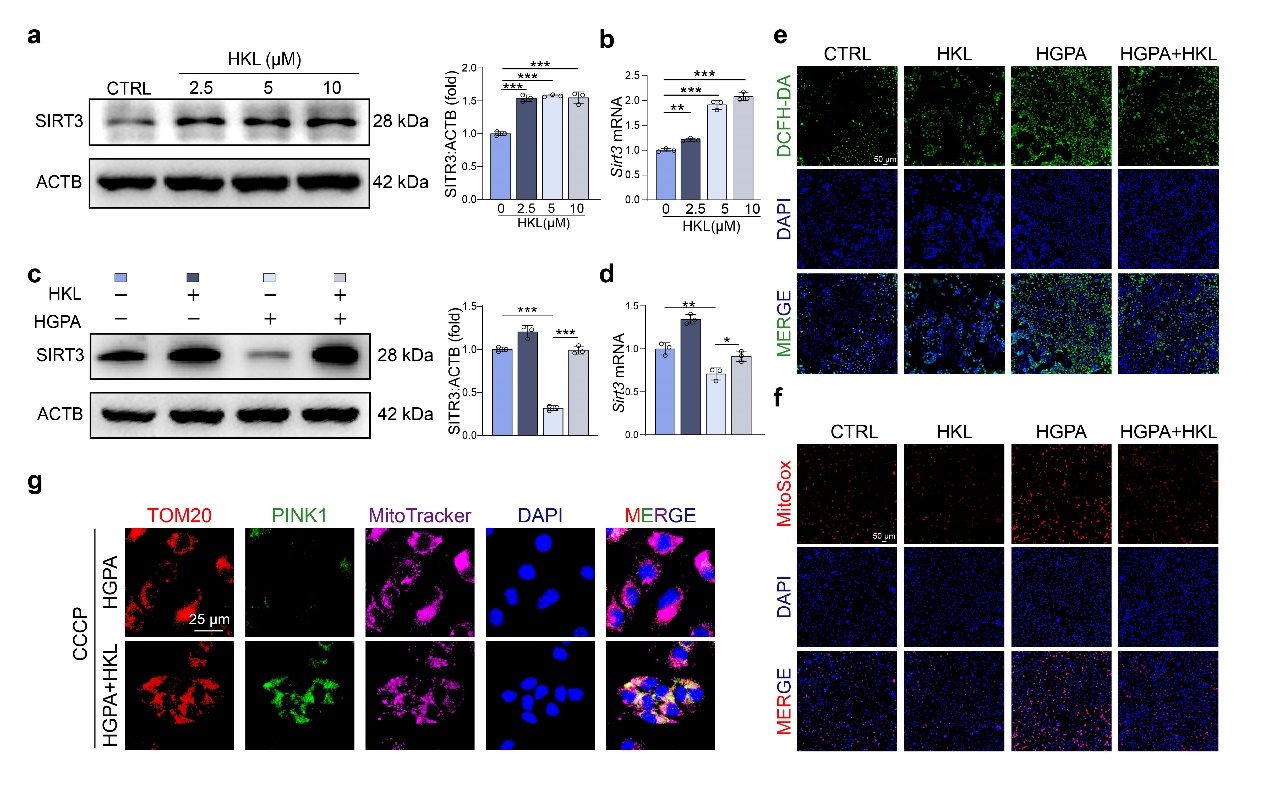
**

**Fig. S2** Activation of SIRT3 alleviates the generation of ROS in MC3T3-E1 cells. (a-d) Relative protein and mRNA expression of SIRT3 in MC3T3-E1 cells treated with HGPA or HKL. (e) The fluorescence images of intracellular ROS using DCFH-DA probe. Scale bar, 50 μm. (f) The fluorescence images of mitochondrial superoxide levels using MitoSox probe. Scale bar, 50 μm. (g) Representative IF staining of TOM20 (red), PINK1 (green), and MitoTracker (deep red) in MC3T3-E1 cells from HGPA and HGPA+HKL groups stimulated by CCCP for 6h. Scale bar, 25 μm. Data presented as mean ± SD. *p＜0.05, **p＜0.01, ***p＜0.001.


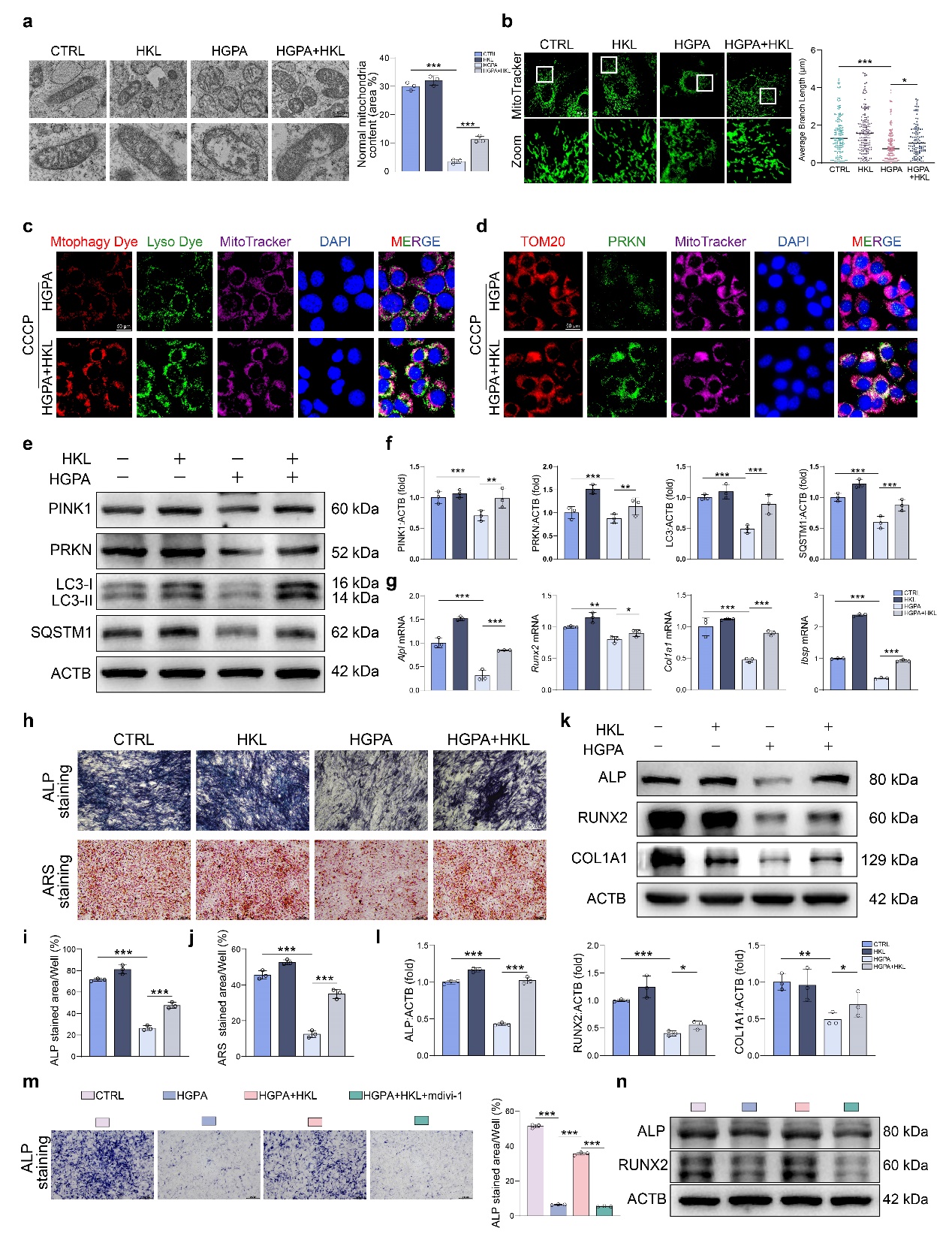


**Fig. S3** Activation of SIRT3 promotes mitophagy and ameliorates osteoblast dysfunction in MC3T3-E1 cells. (a) TEM micrographs and quantification of mitochondrial structures in osteoblasts receiving corresponding treatment. Scale bar, 500 nm. (b) Representative images and quantification of mitochondria in CTRL, HKL, HGPA, and HGPA+HKL groups. Scale bar, 20 μm. (c) Representative images of MitoTracker-labeled mitochondria (deep red), Mtphagy Dye -labeled mitophagy (red), and Lyso Dye-labeled lysosomes (green), of which the co-localization indicates the occurrence of mitophagy. Scale bar, 50 μm. (d) Representative IF staining of TOM20 (red), PRKN (green), and MitoTracker (deep red) in MC3T3-E1 cells from HGPA and HGPA+HKL groups stimulated by CCCP for 6h. Scale bar, 50 μm. (e, f) Western blot images and quantification of mitophagy-related proteins, including PINK1, PRKN, LC3, and SQSTM1 in the above groups. (g) qPCR analysis of *Alpl*, *Runx2*, *Col1a1*, and *Ibsp* genes in 3 independent replicative experiments. (h-j) Representative images and quantification of ALP (7 days) and ARS (21 days) staining of MC3T3-E1 cells from above four group. Scale bar, 200 μm. (k, l) Western blot results and quantitative analysis of ALP, RUNX2, COL1A1 in MC3T3-E1 cells. (m) Representative images and quantification of ALP staining of MC3T3-E1 cells treated with HGPA, HGPA+HKL, and HGPA+mdivi-1. Scale bar, 500 μm. (n) Western blot analysis of ALP and RUNX2 in MC3T3-E1 cells with different intervention. Data presented as mean ± SD. *p＜0.05, **p＜0.01, ***p＜0.001.


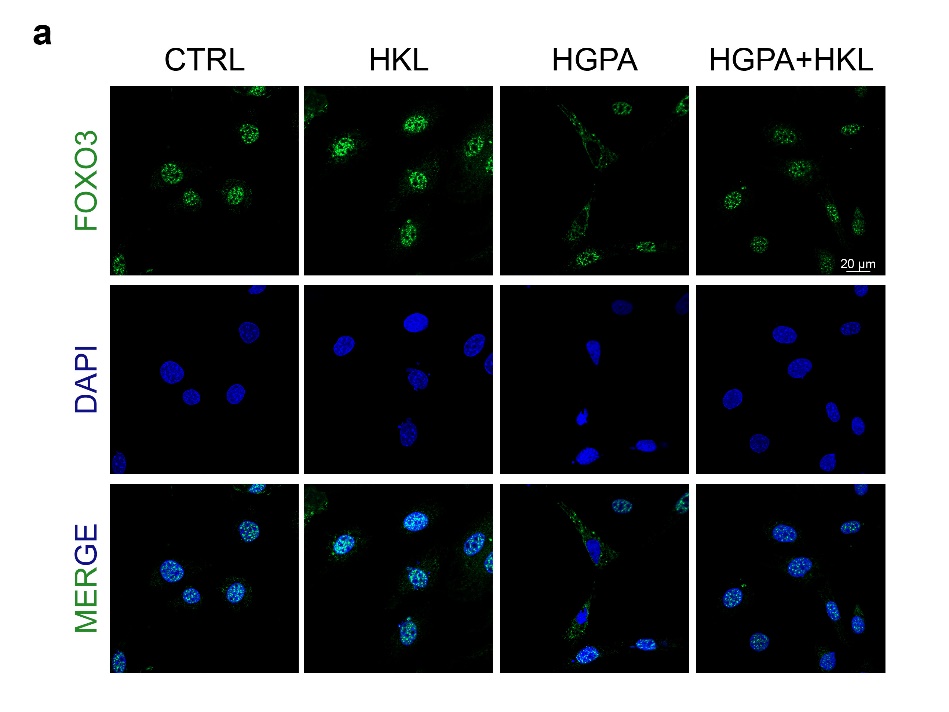


**Fig. S4** SIRT3 deacetylates FOXO3 to regulate *Prkn* gene transcription. (a) Representative IF images of FOXO3 in MC3T3-E1 cells. Scale bar, 20 μm.


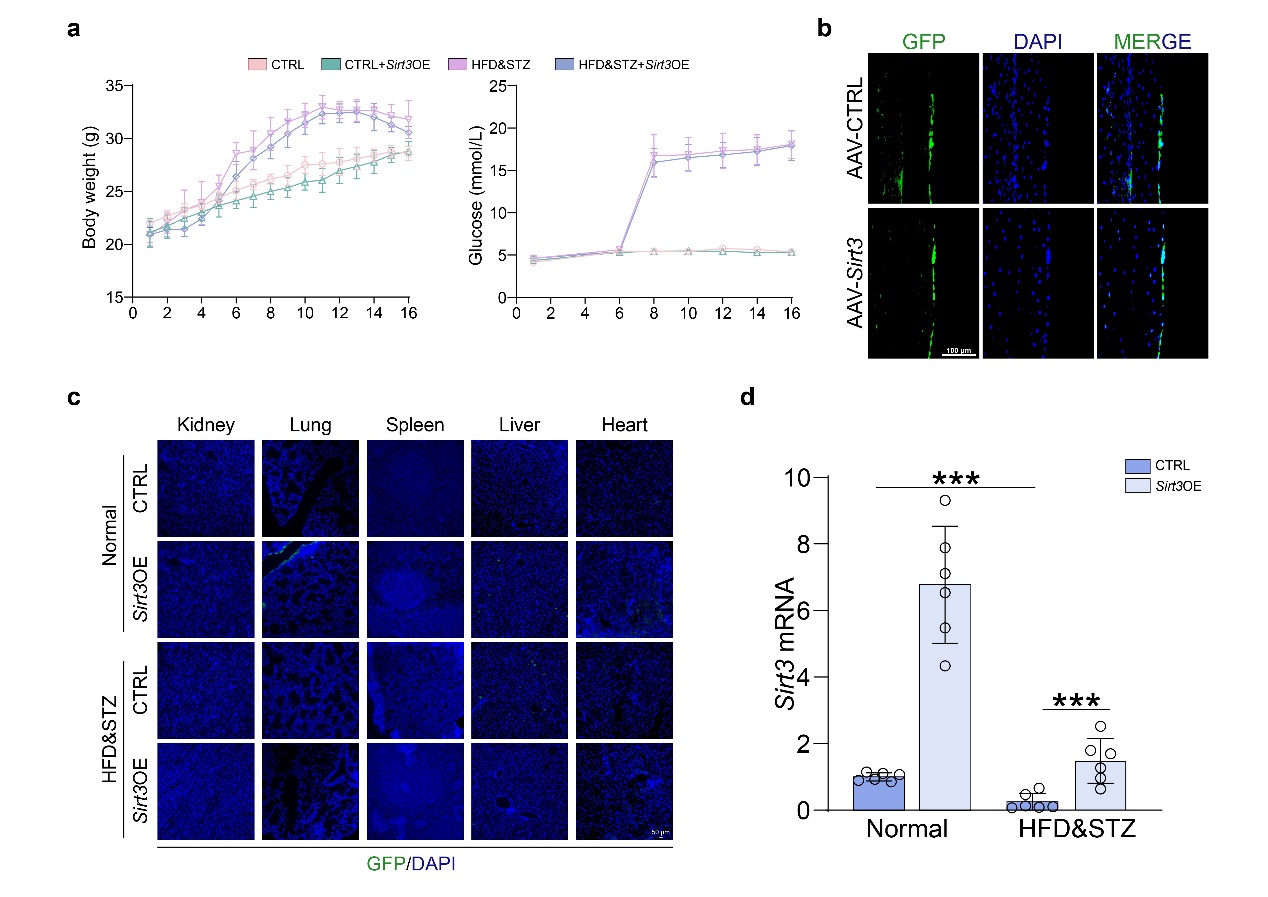


**Fig. S5** Bone-targeted overexpression of *Sirt3* rescues HFD&STZ-induced mitophagy defects. (a) Weekly or biweekly assessment of body weight and fasting blood glucose of normal and HFD&STZ mice receiving AAV-*Ctrl* or AAV-*Sirt3* treatment. (b) Representative images of GFP-expressing cells in the femur. (c) Representative images of GFP expression in major organs. Scale bar, 50 μm. (d) Relative mRNA expression of *Sirt3* genes of femur from normal and HFD&STZ mice receiving AAV-*Ctrl* or AAV-*Sirt3* treatment. Data presented as mean ± SD. **p*＜0.05, ***p*＜0.01, ****p*＜0.001.


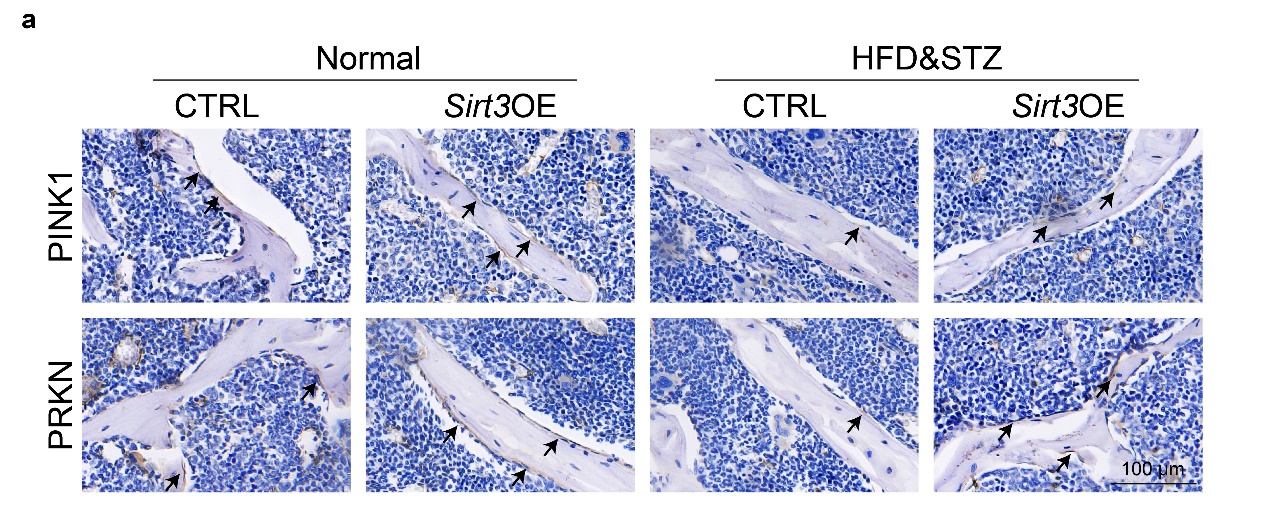


**Fig. S6** *Sirt3*-overexpression rescues HFD&STZ-induced mitophagy defects. (a) IHC staining for SIRT3 of bone sections in CTRL and *Sirt3*OE group from normal and HFD&STZ mice. Scale bar, 100 μm.

**
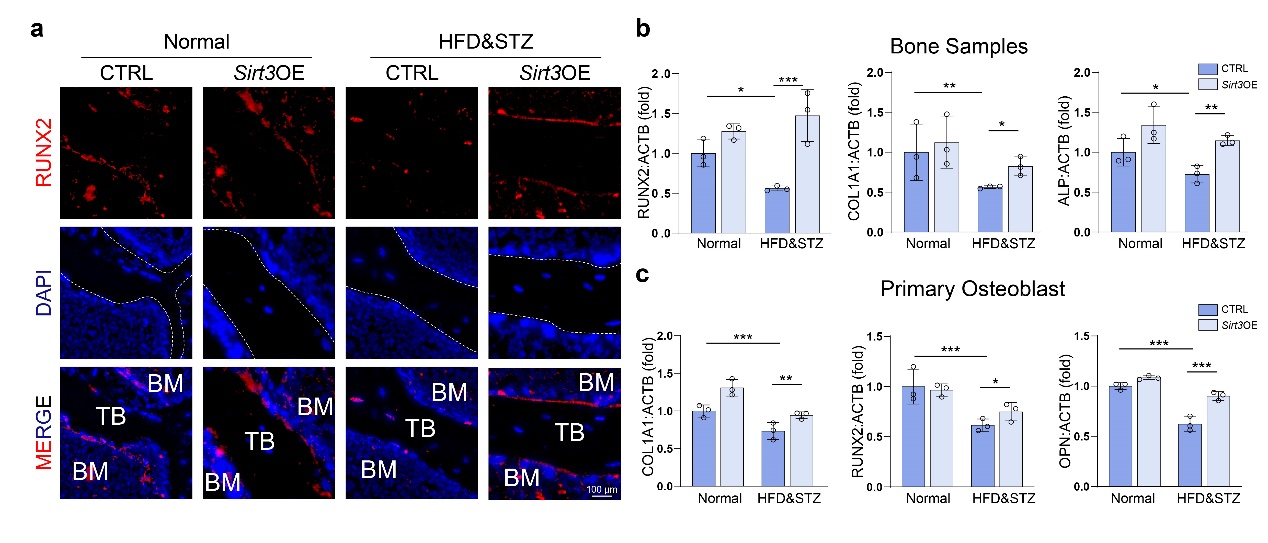
**

**Fig. S7** *Sirt3* overexpression reverses bone loss in HFD&STZ mice. (a) IF staining against RUNX2 of bone tissues. Scale bar, 100 μm. (b) Quantification of RUNX2, COL1A1, and ALP proteins of bone samples. (c) Quantification of COL1A1, RUNX2, and OPN proteins in primary osteoblasts from abovementioned groups. Data presented as mean ± SD. **p*＜0.05, ***p*＜0.01, ****p*＜0.001.

| Gene | Forward | Reverse |
| --- | --- | --- |
| *Alpl* | CCAACTCTTTTGTGCCAGAGA | GGCTACATTGGTGTTGAGCTTTT |
| *Runx2* | CCAACCGAGTCATTTAAGGCT | GCTCACGTCGCTCATCTTG |
| *Spp1* | AGCAAGAAACTCTTCCAAGCAA | GTGAGATTCGTCAGATTCATCCG |
| *Bglap* | CTGACCTCACAGATCCCAAGC | TGGTCTGATAGCTCGTCACAAG |
| *Ibsp* | CAGGGAGGCAGTGACTCTTC | AGTGTGGAAAGTGTGGCGTT |
| *Col1a1* | GCTCCTCTTAGGGGCCACT | CCACGTCTCACCATTGGGG |
| *Lc3* | TTATAGAGCGATACAAGGGGGAG | CGCCGTCTGATTATCTTGATGAG |
| *Prkn* | GGTCCTACAGACAGGGCAATA | CTGGCCTTTCCTCACACCAC |
| *Pink1* | CACACTGTTCCTCGTTATGAAGA | CTTGAGATCCCGATGGGCAAT |
| *Sirt1* | TGATTGGCACCGATCCTCG | CCACAGCGTCATATCATCCAG |
| *Sirt2* | GCGGGTATCCCTGACTTCC | CGTGTCTATGTTCTGCGTGTAG |
| *Sirt3* | GCCCAATGTCACTCACTACTTCCTG | TCCCAGATGCTCTCTCAAGCCCGTC |
| *Sirt4* | GTGGAAGAATAAGAATGAGCGGA | GGCACAAATAACCCCGAGG |
| *Sirt5* | ACCTGTGTCTAGTGGTGGGA | AACCTGAATCTGTCGGTGGC |
| *Sirt6* | AACCCACAAAACATGACCGC | TTTGTCTAGCACGCAGGGTC |
| *Sirt7* | ATTCCTGTCTACAACCGCTCC | CAGGGGAAAGTCTTCAGGGC |
| *Actb* | GGCTGTATTCCCCTCCATCG | CCAGTTGGTAACAATGCCATGT |

Table S1. Primers for quantitative real-time polymerase chain reaction (qPCR)

Table S2. Primers for Cleavage Under Targets and Tagmentation (CUT&Tag) assay

| Gene | Forward | Reverse |
| --- | --- | --- |
| *Prkn* | TCCAAAGGTGTAAGCCTCCC | GCAACTGTCTTCGCTGGTAAC |
| *Spike in* | GCCTTCTTCCCATTTCTGATCC | CACGAATCAGCGGTAAAGGT |
